# Proteomic and metabolomic signatures of rectal tumor discriminate patients with different responses to preoperative radiotherapy

**DOI:** 10.3389/fonc.2024.1323961

**Published:** 2024-02-12

**Authors:** Anna Wojakowska, Lukasz Marczak, Marcin Zeman, Mykola Chekan, Ewa Zembala-Nożyńska, Krzysztof Polanski, Aleksander Strugała, Piotr Widlak, Monika Pietrowska

**Affiliations:** ^1^ Laboratory of Mass Spectrometry, Institute of Bioorganic Chemistry Polish Academy of Sciences, Poznan, Poland; ^2^ The Oncologic and Reconstructive Surgery Clinic, Maria Sklodowska-Curie National Research Institute of Oncology, Gliwice, Poland; ^3^ Department of Pathomorphology, University of Technology, Katowice, Poland; ^4^ Tumor Pathology Department, Maria Sklodowska-Curie National Research Institute of Oncology, Gliwice, Poland; ^5^ Wellcome Sanger Institute, Hinxton, Cambridge, United Kingdom; ^6^ 2nd Department of Radiology, Medical University of Gdańsk, Gdańsk, Poland; ^7^ Center for Translational Research and Molecular Biology of Cancer, Maria Skłodowska-Curie National Research Institute of Oncology, Gliwice, Poland

**Keywords:** rectal cancer, radiotherapy, tissue, metabolomics, proteomics

## Abstract

**Background:**

Neoadjuvant radiotherapy (neo-RT) is widely used in locally advanced rectal cancer (LARC) as a component of radical treatment. Despite the advantages of neo-RT, which typically improves outcomes in LARC patients, the lack of reliable biomarkers that predict response and monitor the efficacy of therapy, can result in the application of unnecessary aggressive therapy affecting patients’ quality of life. Hence, the search for molecular biomarkers for assessing the radio responsiveness of this cancer represents a relevant issue.

**Methods:**

Here, we combined proteomic and metabolomic approaches to identify molecular signatures, which could discriminate LARC tumors with good and poor responses to neo-RT.

**Results:**

The integration of data on differentially accumulated proteins and metabolites made it possible to identify disrupted metabolic pathways and signaling processes connected with response to irradiation, including ketone bodies synthesis and degradation, purine metabolism, energy metabolism, degradation of fatty acid, amino acid metabolism, and focal adhesion. Moreover, we proposed multi-component panels of proteins and metabolites which could serve as a solid base to develop biomarkers for monitoring and predicting the efficacy of preoperative RT in rectal cancer patients.

**Conclusion:**

We proved that an integrated multi-omic approach presents a valid look at the analysis of the global response to cancer treatment from the perspective of metabolomic reprogramming.

## 1 Introduction

Locally advanced rectal cancer (LARC) patients with an increased risk of metastasis or local recurrence (T3-4 or N+) are eligible for neoadjuvant radiotherapy (neo-RT) before surgical resection, which generally leads to a decrease in tumor mass and improves treatment outcomes (1). However, despite the expected benefits of neo-RT, such treatment may not be effective in radioresistant tumors resulting in recurrence in some cases (2). The effectiveness of preoperative RT can be assessed by histopathological analysis of the resected tissue specimen according to tumor regression grading (TRG) system (3). TRG provides valuable prognostic information, yet the actual prediction of tumor regression remains a challenge. Rectal cancer patients are usually monitored using blood tests (e.g., CEA biomarker) and/or imaging (MR, EUS, and CT) to ensure that they remain disease-free and are treated promptly upon relapse. However, in some cases, classical clinical assessment/monitoring tools are insufficient (4). Therefore, the development of novel relevant biomarkers that could be used to predict the effectiveness of neo-RT in LARC patients is eagerly awaited (5). Moreover, there is still a lack of predictive biomarkers of sensitivity/resistance of rectal cancer to RT, which may result in the use of overly aggressive or ineffective therapy with associated negative effects on the quality of life. Therefore, an appropriate prognosis, based on specific predictors, should be the basis for selecting patient groups that require a more aggressive treatment strategy (6, 7).

An improved understanding of the cellular and molecular signaling pathways involved in disease processes, as well as the development of new therapeutic targets, may be made possible by the omics-based methods used to identify molecular risk factors and biomarkers, according to much of the evidence found (8). This could lead to the development of a more potent treatment for LARC patients. Proteomic and metabolomic approaches could be applied to prediction of the response to selected therapeutic strategies and monitoring the progression of disease (9–11). Although RT has been used extensively for a variety of tumors, little progress has been made in predicting and monitoring treatment outcomes after RT (12). There are only a few studies concerning proteomic or metabolomic profiling of tissue or serum/plasma from rectal cancer patients with various RT outcomes (13–18). The majority of these reports only cover neo-chemoradiotherapy’s effects. Moreover, researchers mostly focus on a single protein or panel of a few proteins associated with known radiation effects like DNA repair, cell cycle, cell proliferation, apoptosis, altered metabolism, or immune response. There is lack of a broader, systemic studies combining proteomic and metabolomic approaches to reveal molecular processes and discriminatory molecules correlated with different patient responses to neo-RT in the LARC group. Recently our group applied a multi-omics approach to identify several differentially accumulated proteins and metabolites whose abundances detected in whole serum and serum-derived exosomes differentiated LARC patients with varying neo-RT responses. These molecules were linked to common pathways that are important for the reaction to RT, including energy metabolism, cancer-related signaling pathways, complement activation cascade, platelet functions, and the immune system (19).

In this study, we combined proteomics and metabolomics MS-based approach to identify molecules that could distinguish LARC tumors with various neo-RT responses. We proposed the panel of proteins and metabolites which could be a promising tool for the estimation of radio-responsiveness in patients with rectal cancer. Moreover, we associated differentially accumulated proteins and metabolites with molecular pathways and processes occurring in tumor tissue in response to radiation. Thus, our work provides a holistic view of the rectal cancer tissue response to irradiation from the perspective of metabolic reprogramming.

## 2 Materials and methods

### 2.1 Clinical samples

Tissue samples were taken from 24 LARC patients diagnosed with adenocarcinoma and treated at Maria Skłodowska-Curie National Research Institute of Oncology, Gliwice Branch. All patients were given neo-RT in a total dose of 39-54Gy. Tissue samples were collected between 2012 and 2014, directly during a standard surgical treatment; resected tissue samples were immediately frozen and kept at -80°C until analysis performed in 2020. The histology of three tissue slices (from the edges and center of the studied tissue sample) was assessed by an experienced pathologist for the percentage of tumor cells in each case. TRG assessed routinely in resected tumors reflected the area of residual tumor cells compared to the fibrotic area: TRG0 - complete response/no residual tumor, TRG1 - 10% of residual tumor, TRG2 - 10-50% of residual tumor, and TRG3 - >50% of residual tumor. Depending on the response to the treatment and the presence of tumor cells, collected samples were classified into two groups: good responders (GR) – 12 patients with RT-sensitive tumors (TRG 0-1), and poor responders (PR) - 12 patients with RT-resistant tumors (TRG 2-3). **Table 1** contains the clinicopathological details and disease status for all included patients. Using post-operative material for research purposes was under local Ethics Committee approval no. KB/430-50/12. All tissue donors signed an informed consent form attesting to their voluntarily and consciously taking part.

**Table 1:** Clinical features of study participants with rectal cancer.

		Total *n* (%)	Good Responders *n* (%)	Poor Responders *n* (%)	Difference *p*-Value (test)
**Sex**	Females	11 (45.8)	5 (41.7)	6 (50)	1.0 (Chi2)
	Males	13 (54.2)	7 (58.3)	6 (50)	
**Age (years)**	mean (S.D.) median	66.0 (10.9) 68.5	64 (13.1) 65.0	69 (7.9) 70.5	0.23 (t-test)
**BMI**	mean (SD)	26.2 (4.2)	25.1 (4.0)	27.3 (4.2)	0.19 (t-test)
**Clinical Stage**	II	9 (37.5)	4 (33.3)	5 (41.7)	0.50 (Chi2)
	III	14 (58.3)	8 (66.7)	6 (50.0)	
	IV	1 (4.2)	0 (0.0)	1 (8.3)	
**RT scheme**	39 Gy	11 (45.8)	4 (33.3)	7 (58.3)	0.04 (Chi2)
	42 Gy	8 (33.3)	3 (25.0)	5 (41.7)	
	54 Gy	5 (20.8)	5 (41.7)	0 (13.0)	
**RT** **RT/CT**		12 (50.0) 12 (50.0)	4 (33.3) 8 (66.7)	8 (66.7) 4 (33.3)	0.22 (Chi2)
**Time RT/S (days)**	mean (SD) median	57.0 (22.0) 54.5	56.0 (22.2) 55.0	57.0 (22.6) 44.0	0.88 (t-test)
**Surgery mode**	AR	15 (62.5)	7 (58.3)	8 (66.7)	1.0 (Chi2)
	APR	9 (37.5)	5 (41.7)	4 (33.3)	
**ypT**	0–2	6 (25.0)	4 (33.3)	2 (16.7)	0.64 (Chi2)
	3	18 (75.0)	8 (66.7)	10 (83.3)	
**ypN**	negative	16 (66.7)	9 (75)	7 (58.3)	0.67 (Chi2)
	positive	8 (33.3)	3 (25)	5 (41.7)	
**LNY**	mean (SD)	11.2 (5.1)	10.2 (4.1)	12.0 (4.8)	0.12 (t-test)

BMI, body mass index; RT, neoadjuvant radiotherapy; CT, chemotherapy; Time RT/S, the time from completion of RT to surgery; LNY, node yield; S.D., standard deviation.

### 2.2 Sample preparation for proteomic studies

The ball mill MM400 (Retsch, Germany) was used to grind the whole frozen tissue samples in liquid nitrogen for 45 seconds at 30 Hz. Tissue was lysed in 100 μL of 1% sodium deoxycholate (SDC) in a buffer containing 50mM NH_4_HCO_3_. Following homogenization with a Precellys 24 homogenizer (Bertin Technologies, France), samples were sonicated for 10 minutes in a bath on ice. Then, samples were centrifuged for 10 minutes at 11,000 x g at 4°C and the supernatant was moved to fresh tubes. The amount of isolated protein was measured using Pierce BCA protein assay kit (Thermo Scientific, Rockford, lL, USA) according to the guidelines provided with the product. For in-solution digestion, 10 µl of the sample containing 10 µg of proteins was diluted by adding 15 µl of 50 mM NH_4_HCO_3_ buffer and then reduced with 5.6 mM DTT at 95°C for 5 min. Then, proteins’ thiol groups were alkylated with 5 mM iodoacetamide (IAA) for 20 min at room temperature and in the dark. For digestion, 0.2 µg of sequencing-grade trypsin (Promega) was added to each sample and left overnight at 37°C. Next, 1.5 µg of 10% trifluoroacetic acid (TFA) was added, mixed for 10 minutes, and twice centrifuged for 7 min. at 11,000 x g at 20°C. The purified tryptic peptides were then analyzed by LC–MS/MS.

### 2.3 Mass spectrometry analysis of proteins

A Dionex UltiMate 3000 RSLC nanoLC system combined with a QExactive Orbitrap mass spectrometer (Thermo Fisher Scientific) was used to conduct the proteome analysis. The peptides were separated on an Acclaim PepMap RSLC nanoViper C18 reverse phase column (75 µm x 25 cm, 2 µm particle size) with temperature kept at 30°C and a flow rate of 300 nl/min. The acetonitrile gradient from 4 to 60% in 0.1% formic acid was used in the 190-minute chromatographic program. Mass spectrometry data were acquired using the top 10 DDA approach, MS scans were registered at the resolution of 70,000 (m/z 200) while MS/MS spectra were registered at 17,500 resolution (also at m/z 200) in a positive mode in mass range of 300-2000 *m/z*. Ten most abundant peaks (2 or more charges) were subjected to fragmentation in HCD collision chamber and the collision energy was set for a constant value of 28%. Protein Discoverer 2.2 software (Thermo Fisher Scientific) was used to process the raw data collected during the study. Using the UniProt human database, proteins were identified with an accuracy of 10 ppm for peptide masses and 0.08 Da for fragment ion masses. Methionine oxidation as a dynamic modification and carbamidomethylation of cysteines as a constant modification were set for all searches, and two missed digestion sites per peptide were allowed. Proteins were considered to be identified if the search engine noticed at least two peptides for each protein and a peptide score reached the significance threshold FDR = 0.01 (as determined by the Percolator algorithm). The total ion current (TIC) was used to normalize the identified proteins’ abundance.

### 2.4 Sample preparation for metabolomic studies

50 mg of pulverized tissue was extracted using 200 ul each of hexane, chloroform, methylene chloride, and methanol. The mixture was sonicated for 10 minutes each time after adding organic solvent, then centrifuged for 10 min at 11,000 x g at 4°C, and dried in a vacuum centrifuge. The dried extract was then subjected to derivatization by adding 40 μl of methoxyamine hydrochloride in pyridine (20 mg/ml) and incubated for 1.5h at 37°C. Next, in the second derivatization step, 90 μl of N-Trimethylsilyl-N-methyl trifluoroacetamide was added, and samples were incubated at 37°C for another 30 min. After derivatization, samples were immediately subjected to a GC/MS analysis.

### 2.5 Mass spectrometry analysis of metabolites

The GC-MS system (TRACE 1310 GC oven with TSQ8000 triple quad MS from Thermo Scientific, USA) with a DB-5MS column (30 m 0.25 mm 0.25 m) (J & W Scientific, Agilent Technologies, Palo Alto, California, USA) was used to separate and analyze metabolites. The following conditions were maintained for the gradient during chromatographic separation: 2 minutes at 70°C, followed by 10 minutes at 300°C, at 300°C. The source temperature was set to 250°C, the column interface was maintained at 250°C, and the PTV injector was used to inject the sample with a temperature gradient from 40 to 250°C. The electron ionization energy of the ion source, which operated in the range of 50-850 *m/z*, was set at 70 eV. The mixture of retention indexes (RI) containing alkanes was run before relevant analyses. Raw data files were analyzed using MSDial software (v. 4.92). The correction against the alkane series mixture (C-10-36) was implemented directly in MS Dial to generate the RI for each compound. The 28,220 records in the MSP database from the CompMS site were used to identify small molecules. Metabolite was considered as identified if the similarity index (SI) was above 80%. The following analyses did not include the identified artifacts (alkanes, column bleed, plasticizers, MSTFA, and reagents). Results that had been normalized (by applying the TIC approach) were exported and used in statistical analyses.

### 2.6 Statistical and chemometric analyses

The continuous clinical metadata was compared between GR and PR groups with the T-test, after assessing both groups’ normality (with the Shapiro-Wilk test) and homoscedasticity (with the Levene test). The categorical clinical metadata was compared between groups with the chi-square test of independence. Depending on the normality and homoscedasticity of the data (assessed via the Shapiro-Wilk test and Levene test, respectively), differences in the abundances of proteins and metabolites between independent samples were evaluated using the T-test, Welch test, or U-Mann-Whitney test. Identified compounds were considered as differentially accumulated proteins (DAPs) or differentially accumulated metabolites (DAMs) when the p-value was lower than 0.05. For the false discovery rate correction, the Benjamini-Hochberg protocol was applied in each case. The effect size of 0.5 and 0.8 or 0.3 and 0.5 was considered to be medium and high, respectively, in the effect size analysis using the Hedges’ g or the rank-biserial coefficient of correlation (an effect size equivalent of the U-Mann-Whitney test) (20). The evaluation of pairwise ratios between the specific compounds in the two groups was conducted using the traditional fold change estimator or the Hedges-Lehmann type fold change estimator. All statistical calculations were performed in Python. Normalized data were log-transformed, scaled with a mean-centered factor, and divided by the standard deviation of each variable for chemometric analyses. To show the general sample distribution, Principal Component Analysis (PCA) and Hierarchical Cluster Analysis (HCA) were used. For each compound, a single-feature logistic regression classifier was created. In addition to computing several quality control metrics, leave-one-out validation was carried out. The accuracy was computed as the mean of the TNR (true negative rate—specificity) and TPR (true positive rate—sensitivity) and to be independent of group size. The univariate ROC curve was generated using all of the feature’s data. MetaboAnalyst 5.0 - https://www.metaboanalyst.ca/- was used to carry out the multivariate ROC curve-based exploratory analysis for the prediction of the biomarker panel. ROC curves were created using balanced sub-sampling and Monte Carlo cross-validation (MCCV). The Linear Support Vector Machine (SVM) was used for the analyses, and its built-in algorithm was used to rank the features.

### 2.7 Functional bioinformatics

String ver. 11.5, available at https://string-db.org, was used to analyze proteomic data (21). Hypergeometric testing with Benjamini-Hochberg multiple corrections was used to search for enriched GO terms and Reactome pathways using a list of genes corresponding to DAPs. For protein class annotation, Panther 17.0 Classification System - http://www.pantherdb.org was used. MetaboAnalyst 5.0, available at https://www.metaboanalyst.ca, was used to analyze metabolomic data. The Quantitative Enrichment Analysis (QEA) algorithm was used to identify the metabolic pathways connected to DAMs. The Joint Pathway Analysis tool in MetaboAnalyst 5.0 and Pathview (https://pathview.uncc.edu/) was used to combine and visualize multi-omic data. Integrated pathway analysis, based on the KEGG database, was implemented to carry out this by uploading a list of genes corresponding to DAPs and a list of DAMs with their fold changes. Additionally, the Pearson coefficients were applied to define the correlations between the differentially expressed variables found at both omic levels; p-values 0.05 were considered significant.

## 3 Results

### 3.1 Proteomic and metabolomic profiling of tissue samples

The mass spectrometry-based methods were applied for profiling proteins and metabolites in the tumor tissue of LARC patients who responded differentially to neo-RT. LC-MS/MS label-free approach made it possible to identify 2741 proteins in tissue specimens. The complete list of identified and quantified proteins is presented in **Supplementary Table S1A**, and the major classes of identified proteins are presented in **Supplementary Figure S1A**. Among the most numerous classes of proteins in tumor tissue were RNA metabolism proteins, cytoskeletal proteins, protein modifying enzymes, metabolite interconversion enzymes, and translational proteins. An untargeted GC–MS-based profiling allowed the annotation and relative quantification of 119 metabolites, which are listed in **Supplementary Table S2A**. The most numerous classes of metabolites in tissue samples were amino acids, sugars and derivatives, fatty acids and lipids, carboxylic and hydroxy acids, purines, pyrimidines, and their derivatives (**Supplementary Figure S1B**). Unsupervised clustering of the samples was carried out using the abundances of all identified proteins and metabolites. PCA and HCA performed based on both proteome and metabolome composition of tumor tissue allowed good separation of two groups of samples representing tumors with good and poor responses to neo-RT (**Supplementary Figures S2A–D**).

### 3.2 Proteomic signature of rectal tumor responses to preoperative RT

Among all identified proteins, 1710 showed significantly different (FDR<0.05) abundance between GR and PR, respectively (**Supplementary Table S1A**). Among the most numerous classes of DAPs were RNA metabolism proteins, cytoskeletal proteins, protein modifying enzymes, metabolite interconversion enzymes, and translational proteins (**Supplementary Figure S1C**). Identified DAPs were used to perform supervised clustering of samples (**Supplementary Figure S2E**). Most DAPs were upregulated in the PR group, while only 210 DAPs were upregulated in the GR group. Moreover, 19 DAPs were identified only in the PR group (namely: PROM1, HTATSF1, ND4, IVL, CKMT2, LIG1, BUD31, PTK2, CPSF1, RPS6KA1, ACSM3, ATAD3B, GALNT7, GFM1, COPS8, GTPBP, TRIM2, SPON1, NOX1), while one DAP (INA) was identified only in the GR group.

**Figure 1:**
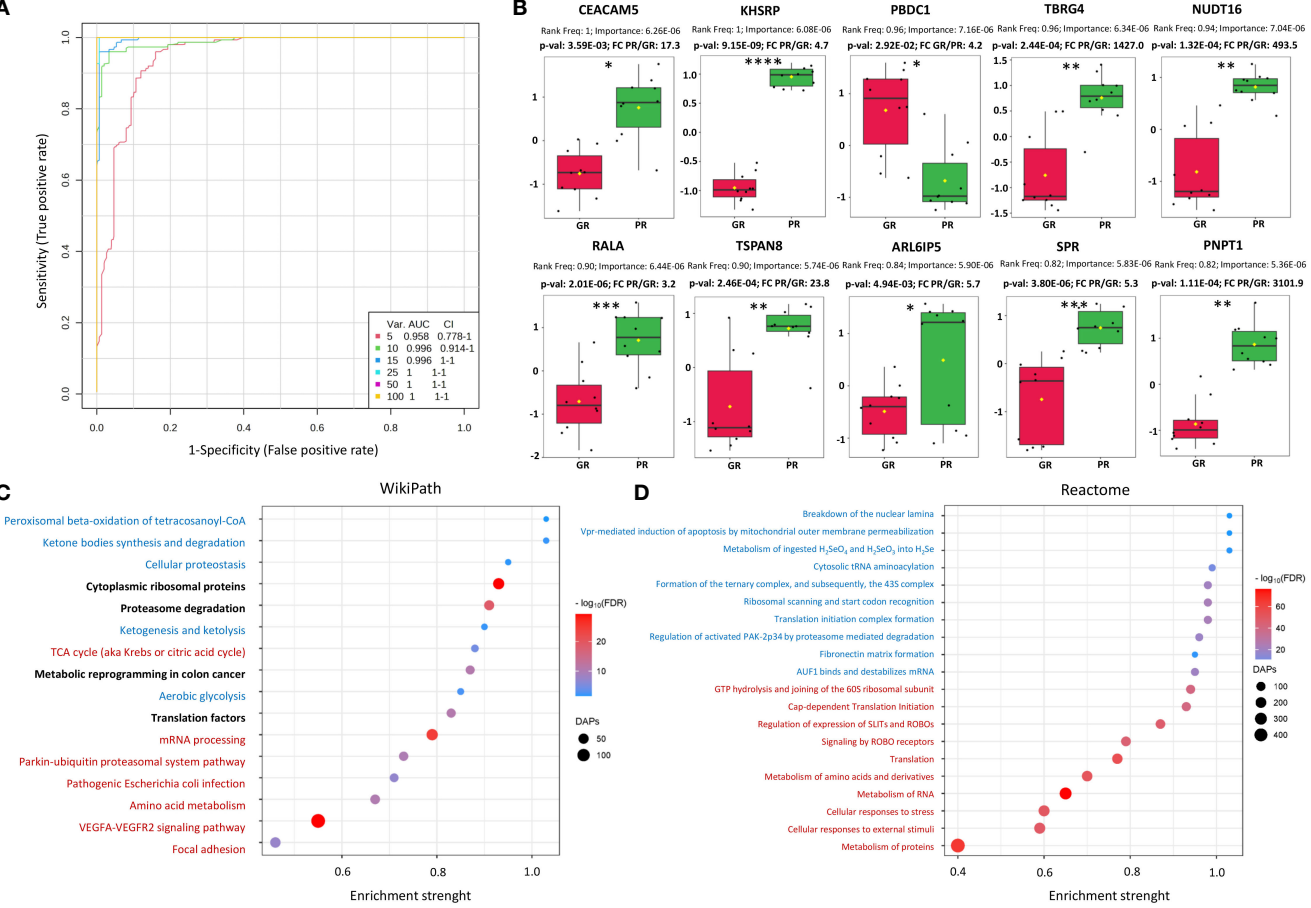
Characterization of proteomic signatures of rectal tumors responses to RT. **(A)** Proteomic-based biomarker prediction by multivariate ROC curve analysis: the classification models based on the top 5, 10, 15, 25, 50, and 100 important proteomic features with their corresponding AUC value; **(B)** The normalized abundances of potential proteomic biomarkers with the highest frequency rank and importance based on the selected classification model, p-values and fold change (FC) values are shown, significance after FDR correction are marked with asterisks according p-adjusted values (p-val_adj_<0.05*, p-val_adj_<0.005**, p-val_adj_<0.0005***, p-val_adj_<0.00005****); **(C, D)** Functional enrichment analysis of DAPs: bubble plots of the TOP20 enriched processes and functions revealed using the Wikipath **(C)** and Reactome **(D)** database, including the top 10 with the largest pathway significance (FDR) (marked in red) and enrichment strength (marked in blue), bolded for both. The highest enriched pathway based on both the significance and pathway impact are bold. The color of the dots represents the p-adjusted values (Benjamini-Hochberg correction), and the size of the dots represents the number of DAPs associated with the GO terms/Reactome pathways.

To further describe the potential of proteins identified in tumor tissues to discriminate patients with different responses to RT, univariate and multivariate classifiers were tested. Based on classical univariate ROC curve analysis, there were 245 proteins (8.9% of all detected proteins) for which a binary classification model (GR vs. PR) was performed with the receiver operating characteristics AUC equal 1 (**Supplementary Table S1B**). Finally, multivariate ROC curve analysis was performed to obtain a panel of potential proteomic biomarkers of response to neo-RT. Proteomic-based biomarker prediction was performed by multivariate ROC curve-based exploratory analysis. The classification models based on the top 5, 10, 15, 25, 50, and 100 important proteomic features with their corresponding AUC values are presented in **Figure 1A**. A model built on 50 features reached a predictive accuracy of 100% (**Supplementary Figures 3A, B**). The predicted class probabilities (average of the cross-validation of 50-mer models) for each sample (GR vs. PR) are presented in **Supplementary Figure 3C**. The top 20 predicted proteomic biomarkers based on how frequently they were selected during cross-validation are shown in **Supplementary Figure 3D**, while the complete list of proposed predictors can be found in **Supplementary Table S1C**. The normalized abundances of the top ten potential proteomic biomarkers with the highest frequency rank and importance based on the 50-mer classification models are presented in **Figure 1B**.

Furthermore, a functional enrichment analysis of DAPs was carried out, which showed a number of significantly overrepresented GO terms linked to them, including 1070 biological processes, 69 molecular functions, and 304 cellular components. Moreover, the KEGG, Reactome, and WikiPathways databases were used to analyze the functional interactions between DAPs (**Supplementary Table S3**). The TOP20 enriched processes (WikiPath) and functions (Reactome) associated with DAPs are presented in **Figures 1C, D**. DAPs’ overrepresented functions and processes were generally connected with focal adhesion, VEGFA-VEGFR2 signaling pathway, metabolism of amino acids and proteins, metabolism of RNA, ribosomal proteins, translation factors, cellular responses to stress, proteasome degradation, ketone bodies, peroxisomal beta-oxidation, energy metabolism (glycolysis and TCA cycle), and metabolic reprogramming in colon cancer. Moreover, significantly overrepresented pathways involved in the immune response (T-cell receptor signaling pathway, antigen processing and presentation, leukocyte, and neutrophil-mediated immunity) were connected with DAPs upregulated in the PR group (**Supplementary Figure S4**). Chosen the most enriched KEGG pathways connected with DAPs, including ribosomal and proteasomal proteins, ECM matrix interaction, proteoglycans in cancer, complement, and coagulation cascades, focal adhesion, and signaling pathways connected with colorectal cancer (VEGF, PI3K-Akt, RAS, WNT, MAPK, NF-KAPPA B) are presented in detail in **Supplementary Figure S5**.

### 3.3 Metabolomic signature of rectal tumor responses to preoperative RT

Among 119 metabolites annotated in rectal tumor tissue, there were 28 DAMs, whose abundances were noticeably (p<0.05) different in PR and GR, respectively (**Supplementary Table S2A**); 7 DAMs after the FDR correction remained significant (namely: ribose 5-phosphate, cytosine, L-carnitine, 4-hydroxybutyric acid, phosphoenolpyruvic acid, inosine, and citric acid). The most numerous classes of DAMs were amino acids, sugars, and their derivatives, carboxylic and hydroxy acids, fatty acids and lipids, and purines/pyrimidines and their derivatives (**Supplementary Figure S1D**). DAMs were used to perform supervised clustering (**Supplementary Figure S2F**). 15 DAMs were upregulated in the GR group, while 13 DAMs were upregulated in the PR group.

**Figure 2:**
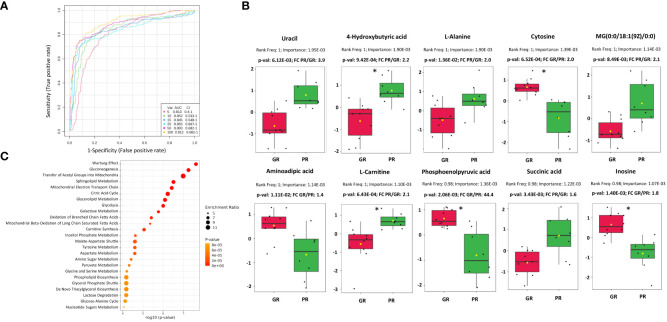
Characterization of metabolomic signatures of rectal tumors responses to RT. **(A)** Metabolomic-based biomarker prediction by multivariate ROC curve analysis: the classification models based on the top 5, 10, 15, 25, 50, and 100 important metabolomic features with their corresponding AUC value; **(B)** The normalized abundances of potential metabolomic biomarkers with the highest frequency rank and importance based on the selected classification model, p-values and fold change (FC) values are shown, significance after FDR correction are marked with asterisks according p-adjusted values (p-val_adj_<0.05) **(C)** Metabolic pathways associated with DAMs based on quantitative enrichment analysis using KEGG database: bubble plot of the TOP 25 enriched metabolite sets.

Univariate classification models tested to classify PR vs. GR samples revealed 24 metabolites with AUC higher or equal to 0.8 (**Supplementary Table S2B**). Multivariate classification models tested based on the top 5, 10, 15, 25, 50, and 100 metabolites with their corresponding AUC values (0.81-0.91) are presented in **Figure 2A**. A model built on 50 features showed the highest predictive accuracy (84.8%) and was selected for further testing (**Supplementary Figures 6A, B**). The class probabilities predicted using this model for each sample are presented in **Supplementary Figure S6C**. The top 20 predicted biomarkers predicted based on how frequently they were chosen for cross-validation are shown in **Supplementary Figure S6D**, while the complete list of proposed predictors can be found in **Supplementary Table S2C**. The normalized abundances of the top 10 potential biomarkers with the highest frequency rank and importance based on the 50-mer classification model are presented in **Figure 2B**.

The Quantitative Enrichment Analysis (QEA) algorithm and the Small Molecule Pathway Database (SMPDB) were used to analyze the functional enrichment of DAMs. (**Supplementary Table S4**). Network view of all significantly enriched pathways (FDR < 0.05) associated with DAMs is shown in **Supplementary Figure S7**, while an overview of the TOP 25 enriched metabolic pathways is presented in **Figure 2C**. The most significant processes associated with DAMs were connected mainly with energy metabolism (e.g., Warburg effect, gluconeogenesis, transfer of acetyl groups into mitochondria, mitochondrial electron transport chain, citric acid cycle, glycolysis), sphingolipid and glycerolipid metabolism, beta-oxidation of fatty acids, carnitine synthesis, inositol metabolism, and amino acids metabolism.

### 3.4 Integration of proteomic and metabolomic features that discriminate between good and poor responders to neo-RT

**Figure 3:**
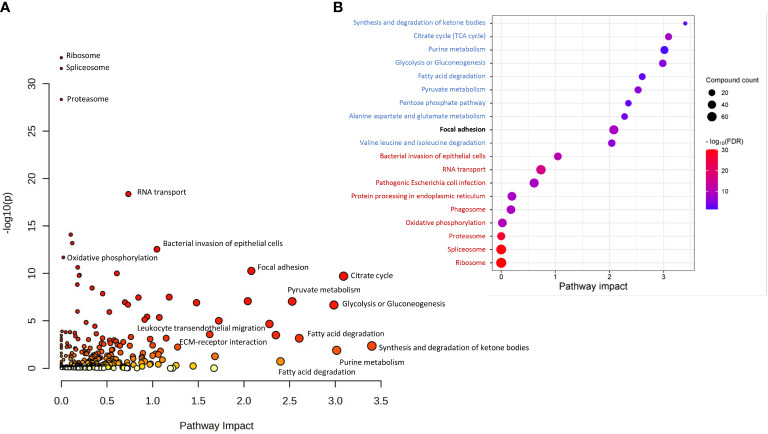
KEGG pathways commonly associated with DAPs and DAMs based on Joint Pathway Analysis. **(A)** all significantly overrepresented pathways (p <0.05); **(B)** The top 20 significantly enriched KEGG pathways, including the top 10 with the largest pathway significance (FDR) (marked in red) and pathway impact (marked in blue). The highest enriched pathway based on both the significance and pathway impact is bold.

Joint Pathway Analysis in MataboAnalyst 5.0 was used to identify common pathways for DAPs and DAMs found in tumors of patients who responded differently to neo-RT. KEGG pathways with the largest pathway significance (p <0.05) connected with DAPs and DAMs are presented in **Figure 3A**. Additionally, the top 20 significant enriched KEGG pathways, including the top 10 with the largest pathway significance (FDR) (marked in red) and pathway impact (marked in blue) are shown in **Figure 3B**. The most significant pathways based on FDR (<4.25E-09) were connected with the ribosome, splicesome, proteasome, oxidative phosphorylation, RNA transport, and bacterial infection. The most enriched pathways based on pathway impact (>2) were synthesis and degradation of ketone bodies, purine metabolism, energy metabolism (TCA cycle, glycolysis, gluconeogenesis, PPP, pyruvate metabolism), fatty acid degradation, and metabolism of amino acids (alanine, aspartate, glutamate, valine, leucine, isoleucine). Focal adhesion was the highest enriched pathway based on both the significance and pathway impact (for details see **Supplementary Table S5**). Components of the most enriched KEGG pathways connected with DAPs and DAMs are presented in detail in **Supplementary Figure S8**.

Furthermore, Pearson’s correlation was used to address any possible relationships between DAPs and DAMs. The investigation turned up several strong correlations (r>0.8) between the variables, which are detailed in **Supplementary Table S6**). For example, CEACAM5 revealed a high positive correlation with a panel of proteins including SLC25A, KHSRP, PNPT1, and MUC 13. On the other hand, there was observed a high negative correlation of CEACAM5 with linoleic acid, oleic acid, phosphoethanolamine, cadaverine, and N-acetyl-aspartic acid. The TOP 25 compounds correlated with CEACAM5 are presented in **Supplementary Figure S9**. Furthermore, known clinical parameters were subjected to the same correlation analysis, which revealed negative correlations of disease stage with linoleic and arachidonic acids as well as positive correlations of the lymph node yield (LNY) with stearic acid and phosphoethanolamine (**Supplementary Table S6**). Because the contribution of the applied RT scheme differed between GR and PR groups (**Table 1**), a putative correlation between the type of RT and the abundance of proteome and metabolome components was also addressed (**Supplementary Table S7**). When the abundance of the Top-10 DAPs (**Figure 1B**) and Top-10 DAMs (**Figure 2B**) in all patients was taken into consideration, components upregulated in the PR group (except alanine) showed a negative correlation while components upregulated in the GR group had a positive correlation with radiation dose, which suggested a potential link between the abundance of differentiating components and response to radiation dose (**Supplementary Figure S10**). Hence, to verify this possibility, the putative associations between the correlation with radiation dose and components’ abundance were analyzed in the PR and GR groups separately to exclude the influence of a hypothetical prognosis factor discriminating between both groups. We found that for the majority of differentiating components, either DAPs or DAMs, the correlations with radiation doses were not statistically significant. Moreover, when significant correlations were found for either PR-upregulated or GR-upregulated components, these correlations observed in each group separately were rather randomly distributed (i.e., either negative or positive) (**Supplementary Table S7**), which further reduced the prognostic significance of radiation dose.

## 4 Discussion

Preoperative (neoadjuvant) RT is a valid strategy for the treatment of LARC. However, the major challenge in this therapeutic approach is cancer radioresistance, which may result in recurrence and metastasis. Therefore, there is a lot of interest in understanding the mechanisms of cancer radio-responsiveness and investigating RT-related biomarkers for the improvement of treatment strategies. Here, for the first time, a combined proteomic and metabolomic approach has been used to reveal a set of molecular components associated with different responses of rectal tumors to neo-RT. DAPs and DAMs were linked to metabolic pathways and signaling processes known to be involved in response to radiation. We observed that the proteome components of tumor tissue have a strong capacity to distinguish between patient samples with different neo-RT responses. A few of the Top 10 potential proteomic biomarkers revealed in our study have been previously identified as compounds associated with colorectal cancer’s response to RT, including CEACAM5, KHSRP, RALA, and TSPAN8. Proteins that regulate glycolysis (PGK1, PGAM1, ENO1, PKM, TKT), ammonia detoxification (GLUD1), and other metabolic pathways (LDHA, GAPDH, MDH2) were reported to be differentially expressed in mouse xenograft colorectal tumor models with different radio- responsiveness (22). In our study, all these proteins (except PGAM1) were elevated in PR. Other DAPs upregulated in PR (CAD, RALB, FAM120A, PSMC2, LRPPRC, PARP1, PSMB5, ANP32B, IMPDH2, XRCC5, TPD52L2, EIFA5A, DDT, GNB1, HDGF, and MYO1C) were associated with metabolic activity in rectal tumor tissue (18). Similarly, a few DAMs upregulated in PR have been previously presented as small molecules associated with radioresistance, including succinic acid and arachidonic acid (23). Succinic acid is an oncometabolite that alters DNA repair through epigenetic regulation and impacts cancer cells’ responses to chemo- and radio-therapy. (24). In our study, we detected significantly elevated levels of both succinic acid and two subunits of succinate dehydrogenase SDHA and SDHB. Furthermore, we observed a significantly elevated accumulation of carnitine (correlated with mitochondrial membrane transporter SLC25A20 - mitochondrial carnitine/acylcarnitine carrier) in PR. Carnitine is essential for shuttling acyl groups through intracellular membranes for fatty acid oxidation (FAO). FAO is essential for the growth and development of many cancers into malignancies. Carnitine is also essential for controlling the acyl-CoA/CoA balance, which controls how carbohydrates and lipids are metabolized. (25). Importantly, we detected a significantly reduced abundance of glutamine in PR and elevated level of proteins connected with glutamine transport and metabolism (SLC1A5 and GLS). In addition to being a crucial component of DNA repair, epigenetic modification, and the reduction of oxidative stress, glutamine metabolism in cancer cells also boosts radioresistance and reduces the effectiveness of radiotherapy and immunotherapy (26). It has been demonstrated that a lack of glutamine increases the epithelial-mesenchymal transition, which in turn promotes the recurrence and metastasis of colorectal cancer (27). Interestingly, different groups of cells present in the tumor may have various nutrient uptake from TME, with glucose being preferentially delivered to immune cells while glutamine and fatty acids are primarily distributed to cancer cells (28). As a result, targeting a single metabolite alone is insufficient to overcome radioresistance because tumor cells and other cells in the TME (including immune system cells) exhibit metabolic heterogeneity (29). Moreover, although the analysis of clinical data revealed statistically significant differences between groups of PR and GR with respect to radiation dose delivered during neo-RT (the contribution of radiation schemes involving higher doses was higher in the GR’ group), radiation dose was barely associated with the abundance of differentiating proteins and metabolites (particularly when the correlations with radiation dose were analyzed in each group separately). Therefore, obtained data suggested that molecular profiles characteristic for GR and PR were not associated directly with response to radiation doses.

**Figure 4:**
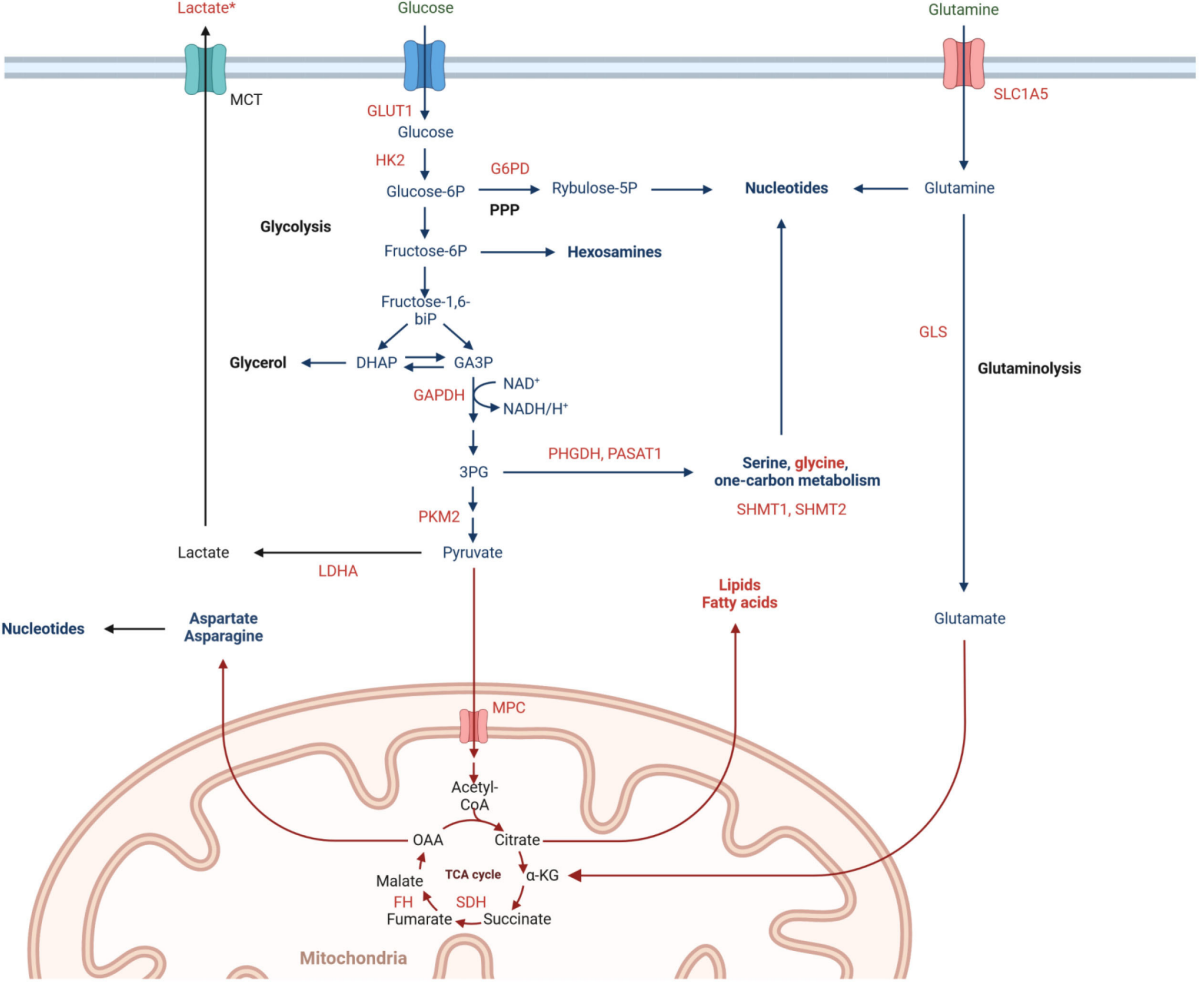
Metabolic pathways involved in rectal cancer radioresistance, including significantly upregulated (marked in red) and downregulated (marked in green) abundances of proteins and metabolites in poor responders tumor tissues. ^*^high effect size.

Obtained proteomic and metabolomic data provided a combination of information on the accumulation of metabolic enzymes and specific metabolites, which enabled to address metabolic reprogramming of rectal cancer. Several identified DAPs and DAMs were functionally linked to alterations in the metabolism of glucose, amino acids, and fatty acids. Tumor cells respond to RT by increasing glucose flux through the upregulation of glycolytic transporters and enzymes, facilitating glucose metabolism including glycolysis, oxidative phosphorylation, and pentose phosphate pathway (PPP) (28). In PR, we observed significantly elevated levels of glycolytic enzymes GLUT1, HK2, GAPDH, PKM2, and LDHA, combined with decreased levels of glucose and increased levels of lactate, which is considered to contribute to radioresistance (30). Metabolic enzymes involved in oxidative phosphorylation (e.g., MPC elevated in poor responders) and the integrity of mitochondrial function are crucial for cancer radioresistance, while the activity of 6PGD (a component of PPP) enhances the production of NAPDH and nucleotides that promote tumor growth and radioresistance (31). Additionally, tumor cells respond to RT by increasing the metabolism of amino acids like glutamine, serine, and glycine, which provide the biomacromolecules and other materials needed for the production of nucleotides and energy, extending the survival of cancer cells. Glutamine is transported into the cell by SLC1A5 and converted to glutamate by mitochondrial glutaminase (GLS). It has been shown that radiation increases the GLS activity contributing to radioresistance (32). In our study, we detected significantly elevated levels of SLC1A5 and GLS in PR, while the abundance of glutamine was decreased. Moreover, aberrantly activated glycolysis permits tumor cells to indirectly enhance serine/glycine metabolism, increasing one-carbon metabolic flux and facilitating the proliferation of tumor cells and radioresistance (33). Here we detected in PR elevated levels of proteins involved in serine/glycine and one-carbon metabolism (PHGDH, PASAT1, SHMT). Moreover, enhanced accumulation of glycine and serine was also observed. Furthermore, cancer cells may develop radioresistance via reprogramming of lipid metabolism. We detected in PR elevated levels of enzymes (COX-2, ACSS2, FDPS, FASN, ACAT2, ACLY, SLC12A2) and metabolites (arachidonic acid and carnitine) involved in lipid metabolism, which have been linked to radioresistance of cancer cells (29). The major mechanism that enables the development of tumor radioresistance is DNA damage repair, which needs a significant nucleotide accumulation. Hence, activated glucose and amino acid metabolism that provide sufficient substrates and energy for the synthesis of pyrimidines and purines are linked to the survival of irradiated tumor cells. On the other hand, enzymes involved in the *de novo* nucleotide synthesis pathway (e.g. IMPDH) have emerged as targets for radiosensitization (29). Here we detected in PR elevated levels of inosine monophosphate dehydrogenase, while the abundance of inosine was reduced. In general, we concluded that molecular profiles characteristic of PR fit the metabolic reprogramming state that enhances tumor radioresistance, which involves the increased metabolic flux of glucose, fatty acids, lipids, and amino acids (especially glutamine), thus supplying sufficient energy and substrates for DNA damage repair. These metabolic pathways likely involved in the development of tumor radioresistance in the group of PR are illustrated schematically in **Figure 4**.

In conclusion, here we applied a combined MS-based proteomic and metabolomic approach for the identification in tumor tissue of molecules that discriminate LARCs differentially responded to neo-RT. This revealed molecular pathways and processes associated with DAPs and DAMs, which were linked to favorable and unfavorable responses to the treatment. These included several pathways involved in cellular metabolism and metabolic reprogramming, including energy metabolism, ketone bodies metabolism, fatty acid degradation, metabolism of amino acids and purines, which appeared to play a vital role in the radioresistance of tumors. Hence, our study revealed that multi-component panels of proteins and metabolites may serve as a solid base to develop biomarkers for monitoring and predicting the efficacy of preoperative RT in this group of patients as well as serve as therapeutic targets acting in combination with RT. However, our study has some limitations that could be addressed in future research. First, to confirm that the observed signatures were specific for cancer cells not to “normal” cells present in the tumor stroma, additional analyses using isolated cancer cells (e.g., by microdissection) might be instructive. Moreover, to validate the actual predictive potential of proposed signatures, their components should be analyzed in tissue material (e.g., in biopsies) before preoperative RT. Nevertheless, this explorative study provides proof of concept that molecular components of tumors that are associated with differentiated radio-responsiveness of rectal cancer could identified by the metabolomics and proteomics approaches.

## Data availability statement

The datasets presented in this study can be found in online repositories. The mass spectrometry proteomics data have been deposited to the ProteomeXchange Consortium via the PRIDE partner repository with the dataset identifier PXD048647 and DOI:10.6019/PXD048647. The mass spectrometry proteomics data are available at the NIH Common Fund's National Metabolomics Data Repository (NMDR) website, the Metabolomics Workbench, https://www.metabolomicsworkbench.org where it has been assigned Study ID ST003045. The data can be accessed directly via its Project DOI: http://dx.doi.org/10.21228/M8S14W. This work is supported by NIH grant U2C-DK119886.

## Ethics statement

The studies involving humans were approved by Maria Skłodowska-Curie National Research Institute of Oncology Gliwice Branch Ethics Committee. The studies were conducted in accordance with the local legislation and institutional requirements. The participants provided their written informed consent to participate in this study.

## Author contributions

AW: Conceptualization, Data curation, Formal Analysis, Funding acquisition, Investigation, Methodology, Project administration, Supervision, Writing – original draft, Writing – review & editing. LM: Investigation, Methodology, Writing – review & editing. MZ: Resources, Writing – review & editing. MC: Resources, Writing – review & editing. EZ-N: Resources, Writing – review & editing. KP: Formal Analysis, Writing – review & editing. AS: Investigation, Writing – review & editing. PW: Writing – review & editing. MP: Conceptualization, Writing – review & editing.

## Supplementary material

Supplementary Figure 1 Contribution of identified proteins **(A)**, annotated metabolites **(B)**, DAPs **(C)**, and DAMs **(D)** to different classes.

Supplementary Figure 2 Clustering of RC patients based on levels of proteins and metabolites detected in tissue samples. Showed are PCA score plots **(A, B)** and dendrograms resulting from HCA **(C, D)**; a number of samples that were used only for proteomic or metabolomic profiling are marked in asterisk). The colors navy blue and pink, respectively, indicate GR and PR samples. Hierarchical supervised clustering was performed based on levels of DAPs **(E)** and DAMs **(F)**. ^*^sample appearing only in proteomic or metabolomic analysis.

Supplementary Figure 3 Biomarker prediction by multivariate ROC curve analysis based on proteomic features. **(A)** - The predictive accuracies of 6 different biomarker models; For the 50-feature panel, the red dot indicates the highest accuracy.; **(B)** - ROC curve for a chosen biomarker model with the highest accuracy; **(C)** -The predicted class probabilities for each sample (GR vs. PR); **(D)** - The top 20 potential proteomic biomarkers predicted based on how frequently they were chosen for cross-validation.

Supplementary Figure 4 Overrepresented pathways involved in the immune response connected with DAPs upregulated in PR. **(A)** - bubble plots of the TOP12 enriched immunological processes and functions revealed using the GO terms; **(B)** - chosen significantly overrepresented pathways involved in the immune response.

Supplementary Figure 5 Chosen the most enriched KEGG pathways connected with DAPs. **(A)** - Ribosome; **(B)** - Proteasome; **(C)** - ECM-receptor interaction; **(D)** - Proteoglycans in cancer; **(E)** - Focal adhesion; **(F)** - Complement and coagulation cascade; **(G)** - Colorectal cancer; **(H)** - PI3K-AKT signaling pathway; **(I)** - WNT signaling pathway; **(J)** - MAPK signaling pathway; **(K)** - RAS signaling pathway; **(L)** - VEGF signaling pathway; **(M)** - NF-KAPPA B signaling pathway.

Supplementary Figure 6 Biomarker prediction by multivariate ROC curve analysis based on metabolomic features. **(A)** - The predictive accuracies of 6 different biomarker models; For the 50-feature panel, the red dot indicates the highest accuracy.; **(B)** - ROC curve for a chosen biomarker model with the highest accuracy; **(C)** - The predicted class probabilities for each sample (GR vs. PR); **(D)** - The top 20 potential proteomic biomarkers predicted based on how frequently they were chosen for cross-validation.

Supplementary Figure 7 Network view of significantly enriched metabolic pathways (FDR < 0.05) associated with DAMs based on quantitative enrichment analysis using KEGG database.

Supplementary Figure 8 Chosen the most enriched KEGG pathways connected with DAPs and DAMs. **(A)** - Glycolysis/gluconeogenesis; **(B)** - Pyruvate metabolism; **(C)** - Pentose phosphate pathway; **(D)** - Citrate cycle; **(E)** - Purine metabolism; **(F)** - Pyrimidine metabolism; **(G)** - Fatty acid degradation.

Supplementary Figure 9 The TOP 25 compounds correlated with CEACAM5 based on Pearson’s correlation analysis.

Supplementary Figure 10 The results of Pearson’s correlation analysis of the Top-10 DAPs and DAMs correlated with radiation dose in all patient groups.
