# Supplementary material for: Proteomic and metabolomic signatures of rectal tumor discriminate patients with different responses to preoperative radiotherapy
Source: Front Oncol. 2024 Feb 12;14:1323961. doi: 10.3389/fonc.2024.1323961 (PMC10896604; doi:10.3389/fonc.2024.1323961)
Supplement: Supplementary Figure 1 — Contribution of identified proteins (A), annotated metabolites (B), DAPs (C), and DAMs (D) to different classes. [file Image_1.pdf]

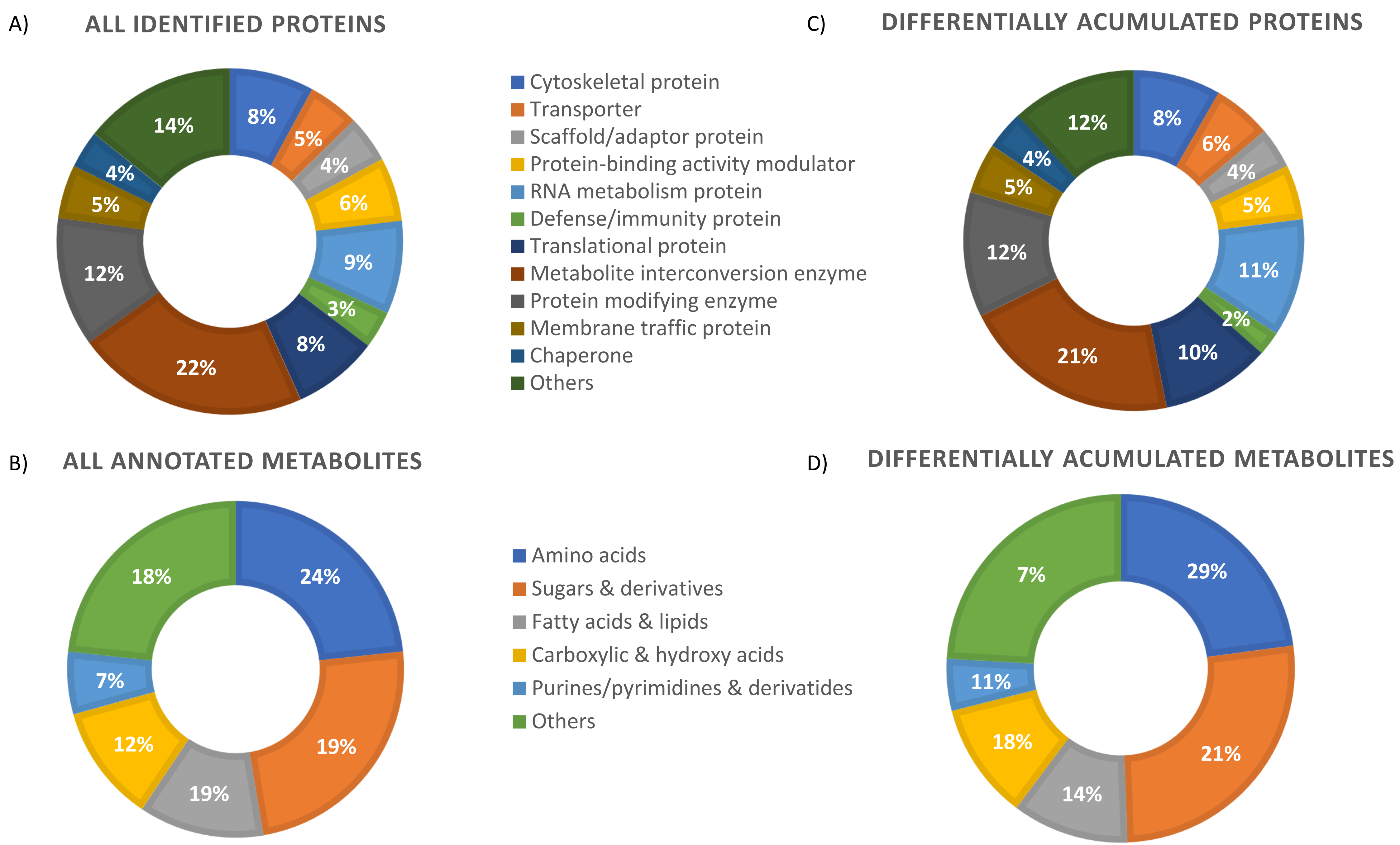
A)
ALL IDENTIFIED PROTEINS
8%
5%
4%
6%
9%
3%
8%
22%
12%
5%
4%
14%
Cytoskeletal protein
Transporter
Scaffold/adaptor protein
Protein-binding activity modulator
RNA metabolism protein
Defense/immunity protein
Translational protein
Metabolite interconversion enzyme
Protein modifying enzyme
Membrane traffic protein
Chaperone
Others
C)
DIFFERENTIALLY ACUMULATED PROTEINS
8%
6%
4%
5%
11%
2%
10%
21%
12%
5%
4%
12%
B)
ALL ANNOTATED METABOLITES
24%
19%
19%
12%
7%
18%
Amino acids
Sugars & derivatives
Fatty acids & lipids
Carboxylic & hydroxy acids
Purines/pyrimidines & derivatides
Others
D)
DIFFERENTIALLY ACUMULATED METABOLITES
29%
21%
14%
18%
11%
7%
