# Supplementary figures and images for: Proteomic and metabolomic signatures of rectal tumor discriminate patients with different responses to preoperative radiotherapy
Source: Front Oncol. 2024 Feb 12;14:1323961. doi: 10.3389/fonc.2024.1323961 (PMC10896604; doi:10.3389/fonc.2024.1323961)

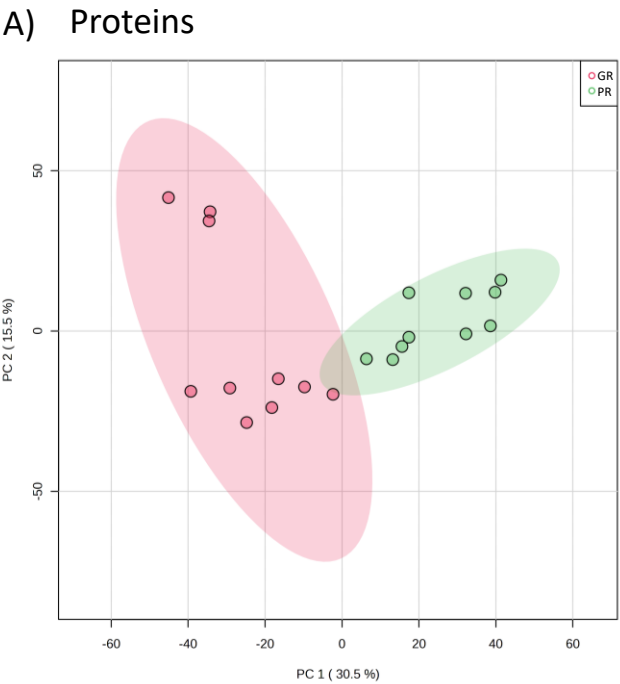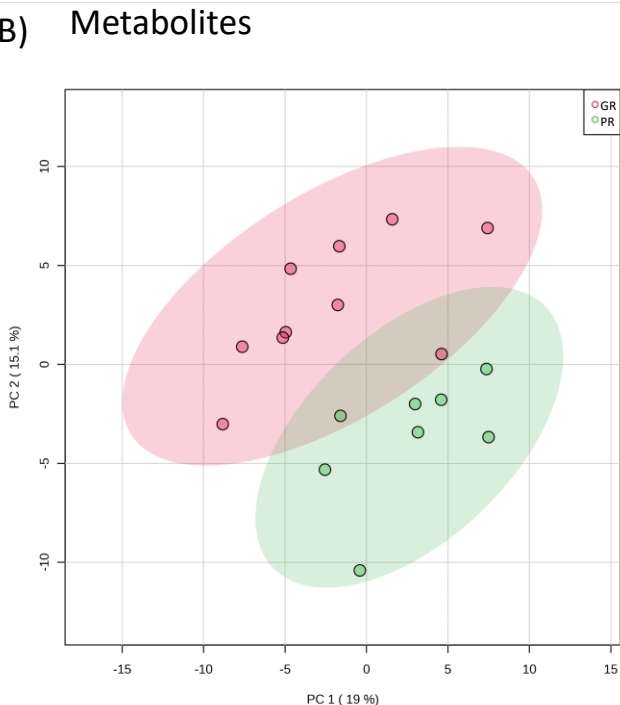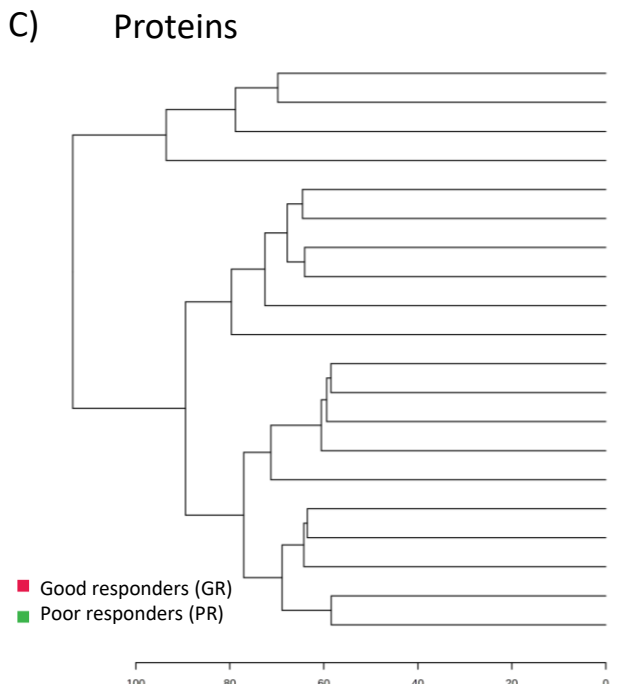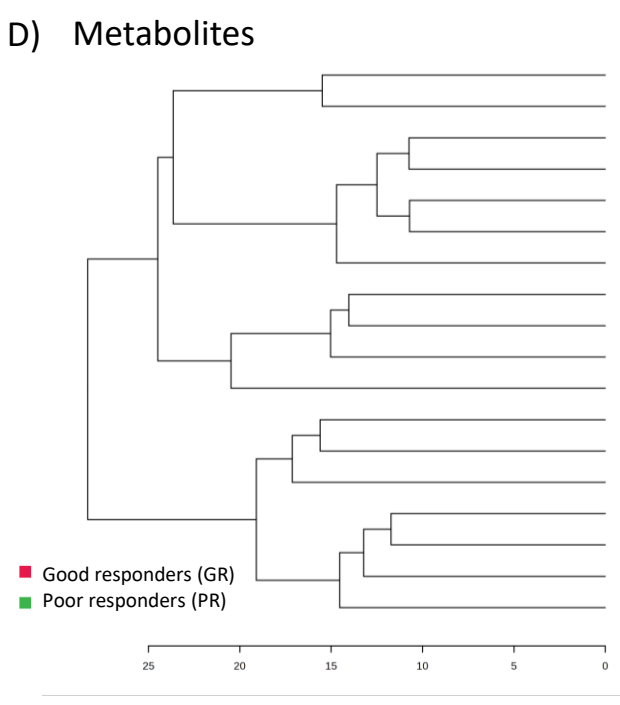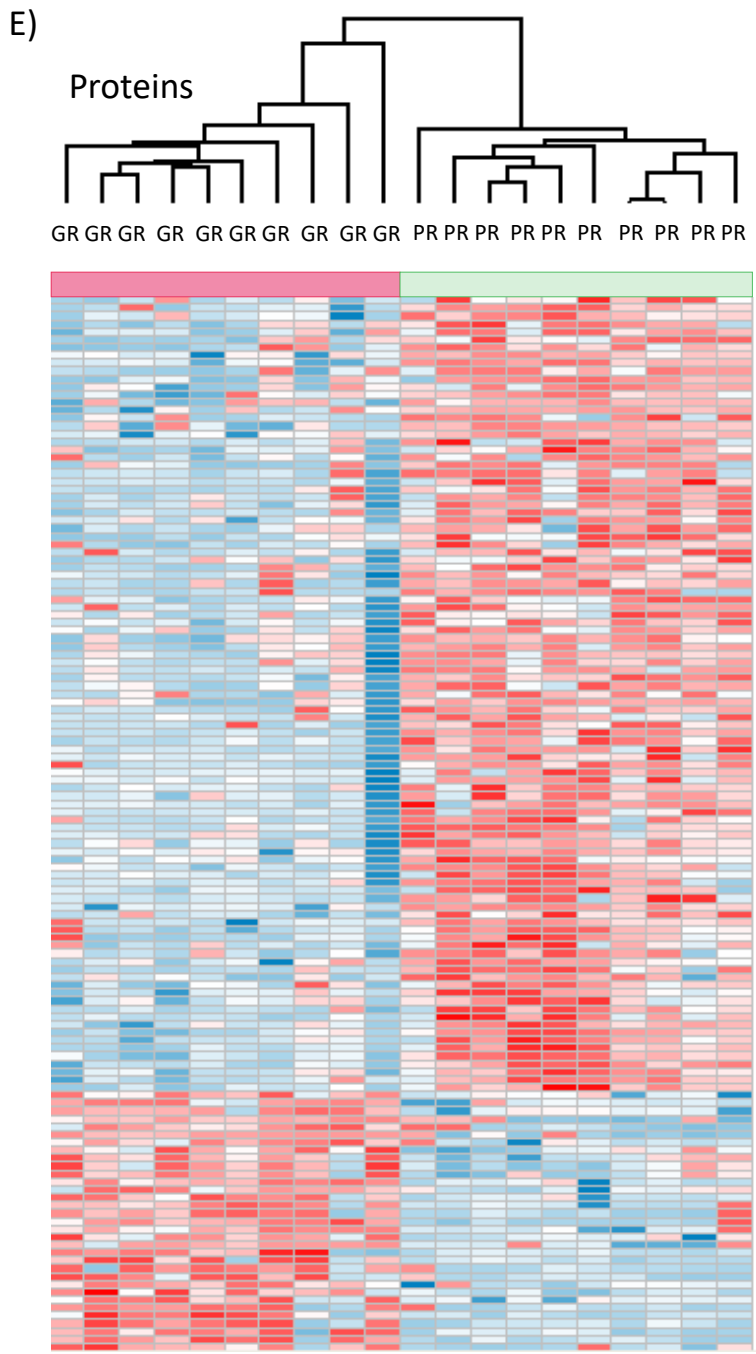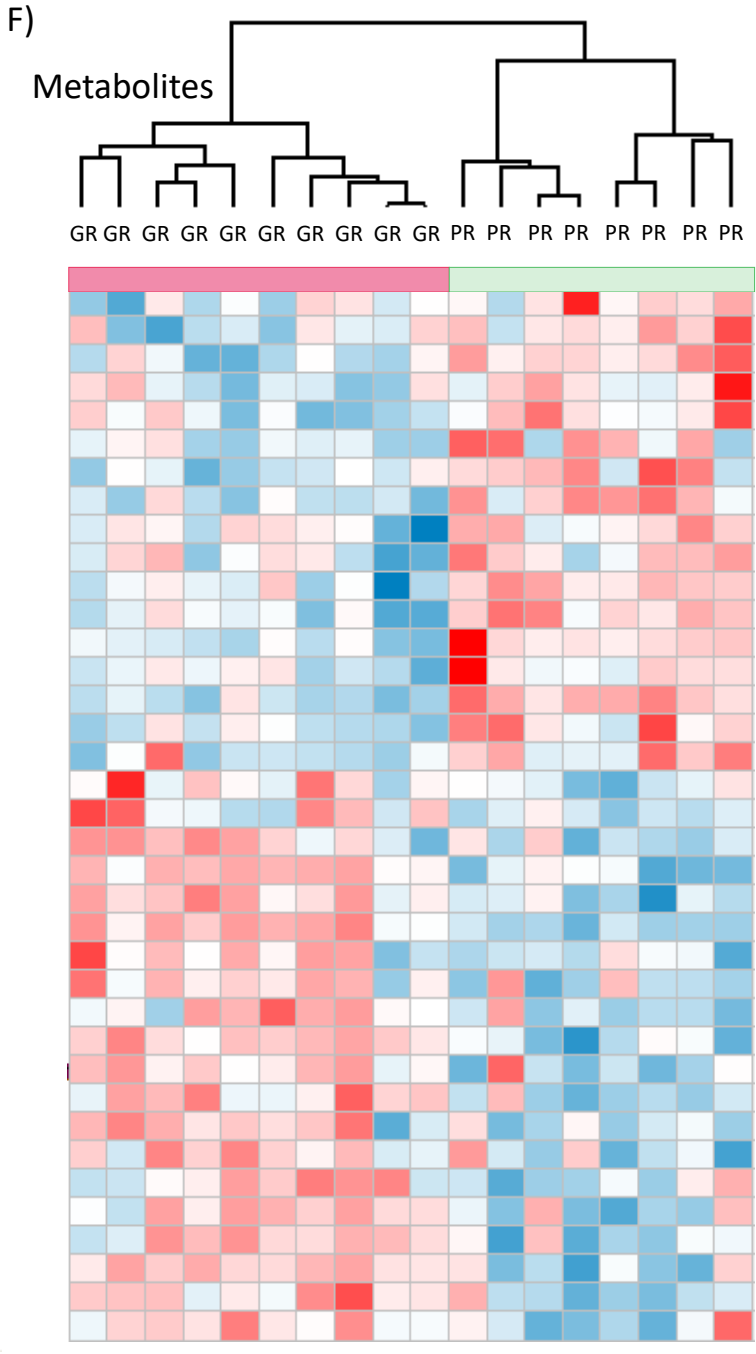

Supplement: Supplementary Figure 2 — Clustering of RC patients based on levels of proteins and metabolites detected in tissue samples. Showed are PCA score plots (A, B) and dendrograms resulting from HCA (C, D); a number of samples that were used only for proteomic or metabolomic profiling are marked in asterisk). The colors navy blue and pink, respectively, indicate GR and PR samples. Hierarchical supervised clustering was performed based on levels of DAPs (E) and DAMs (F). *sample appearing only in proteomic or metabolomic analysis. [file Image_2.pdf]

C)

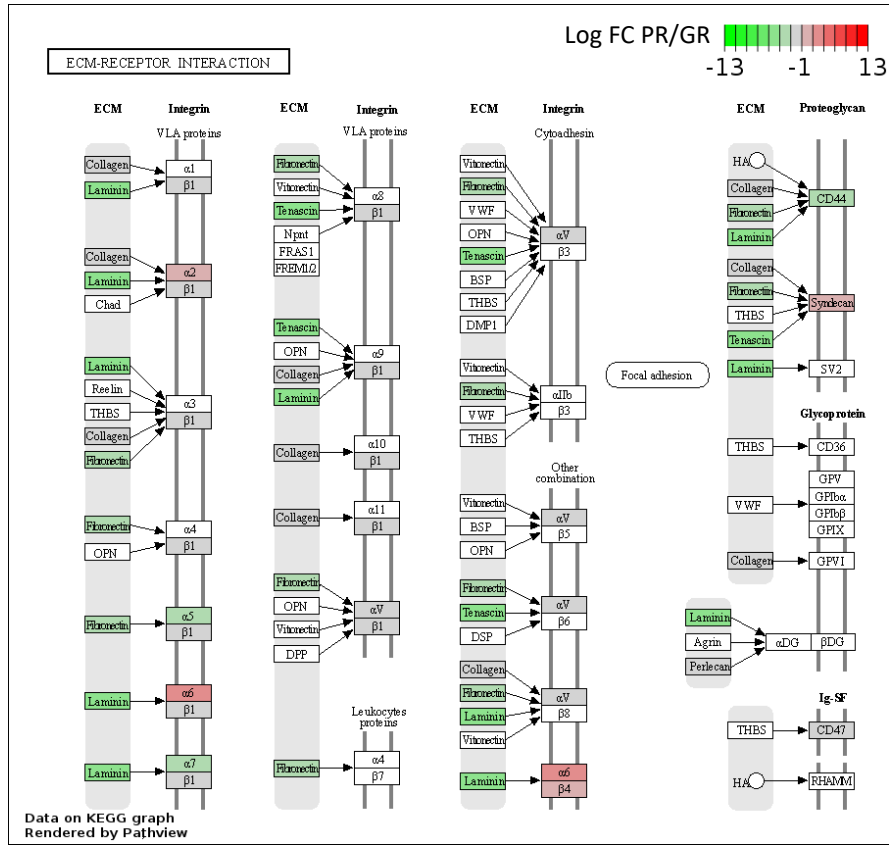

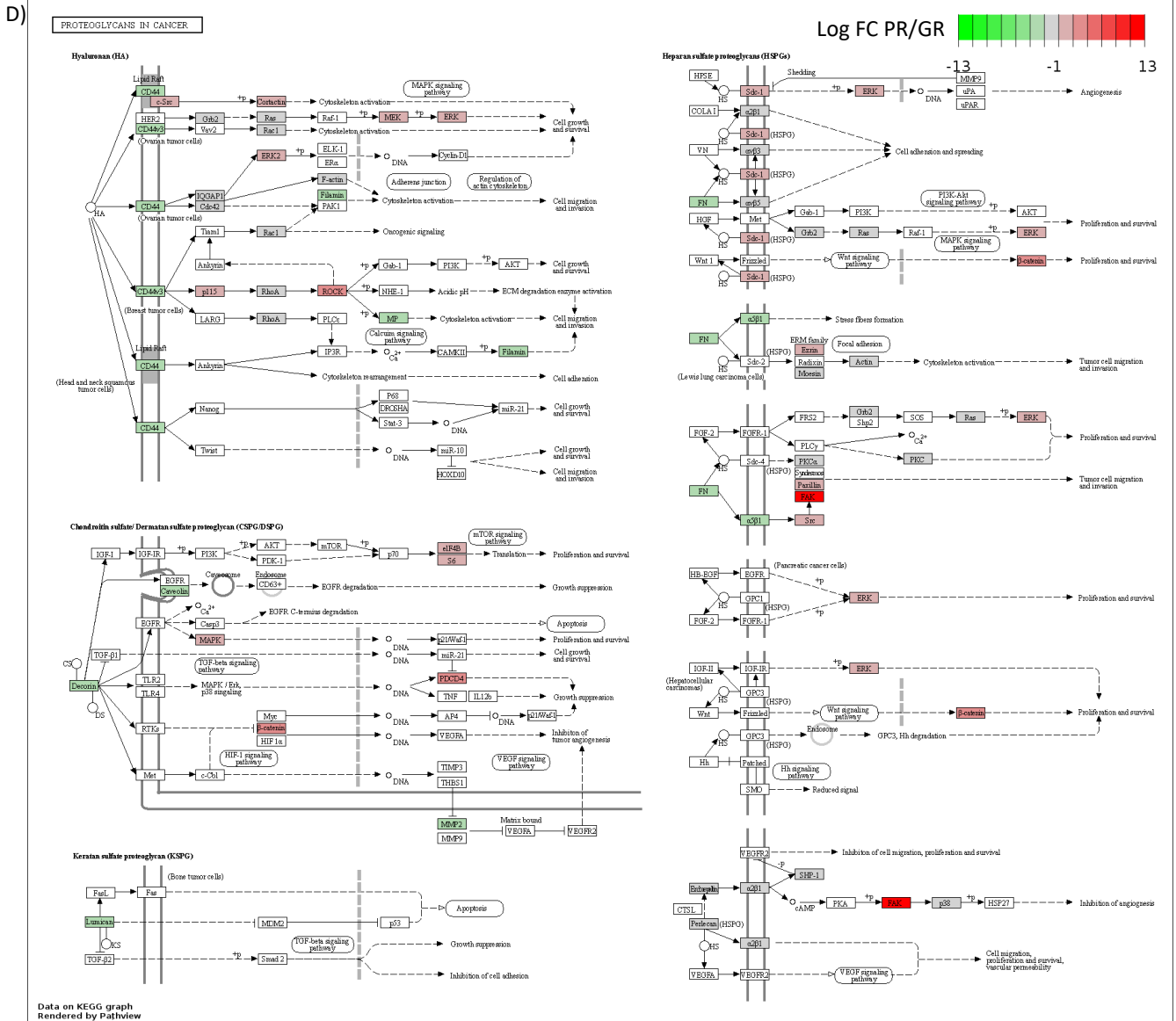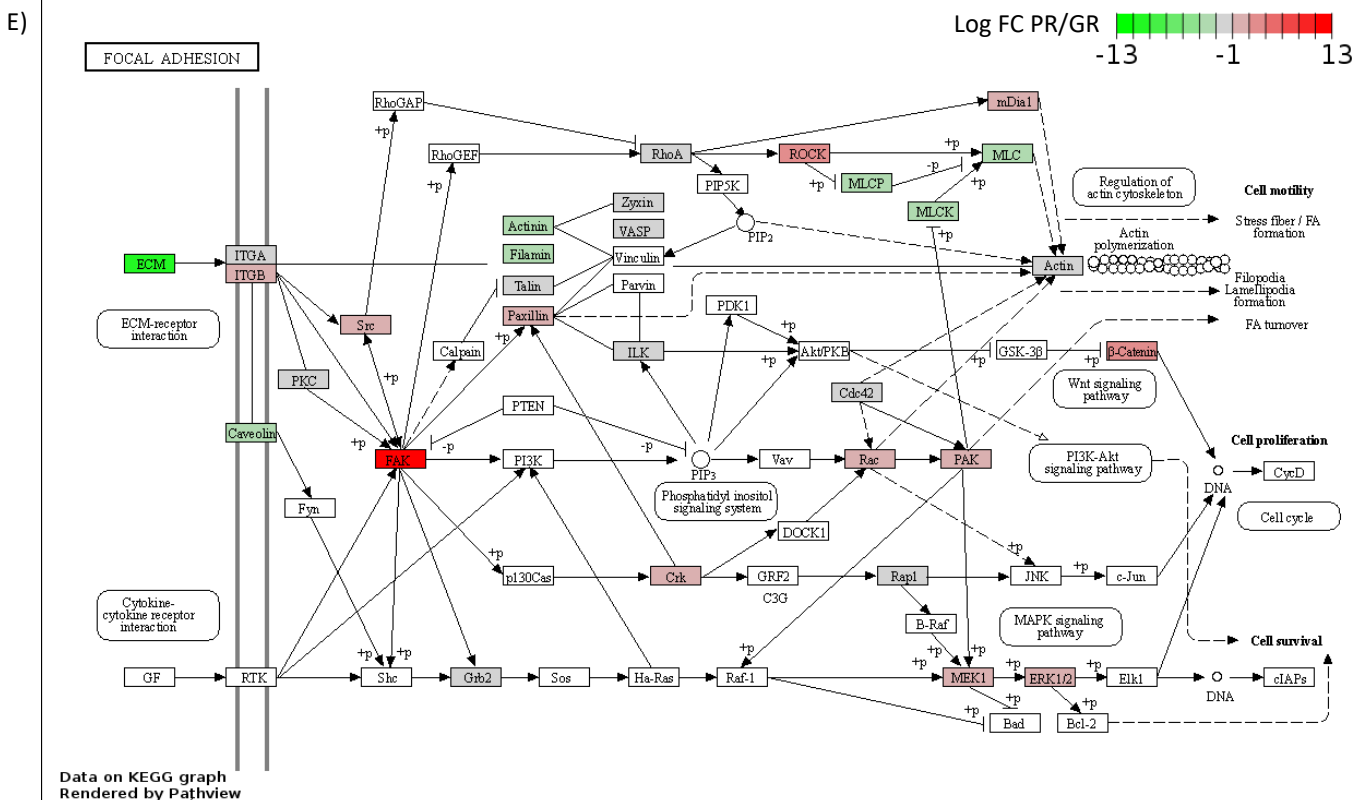

F)

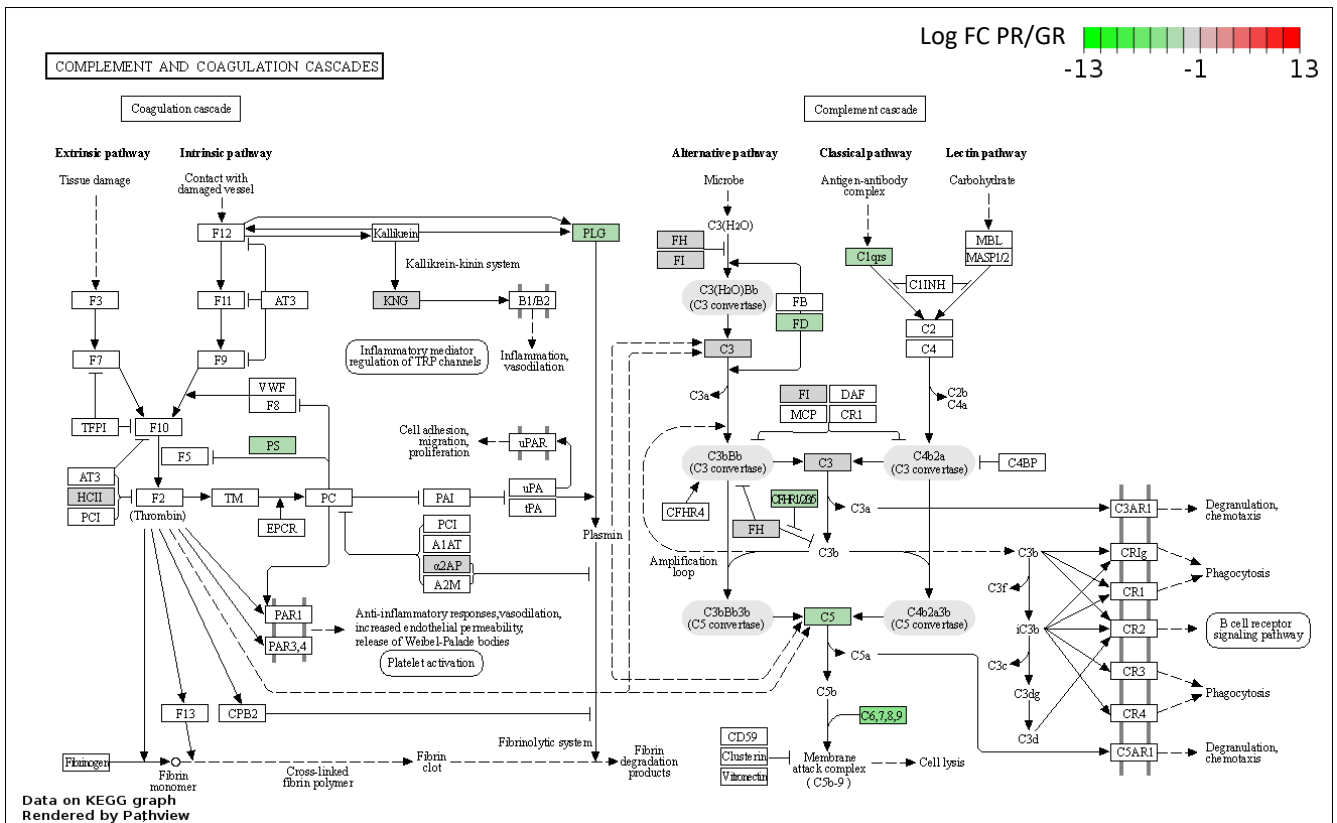

G)

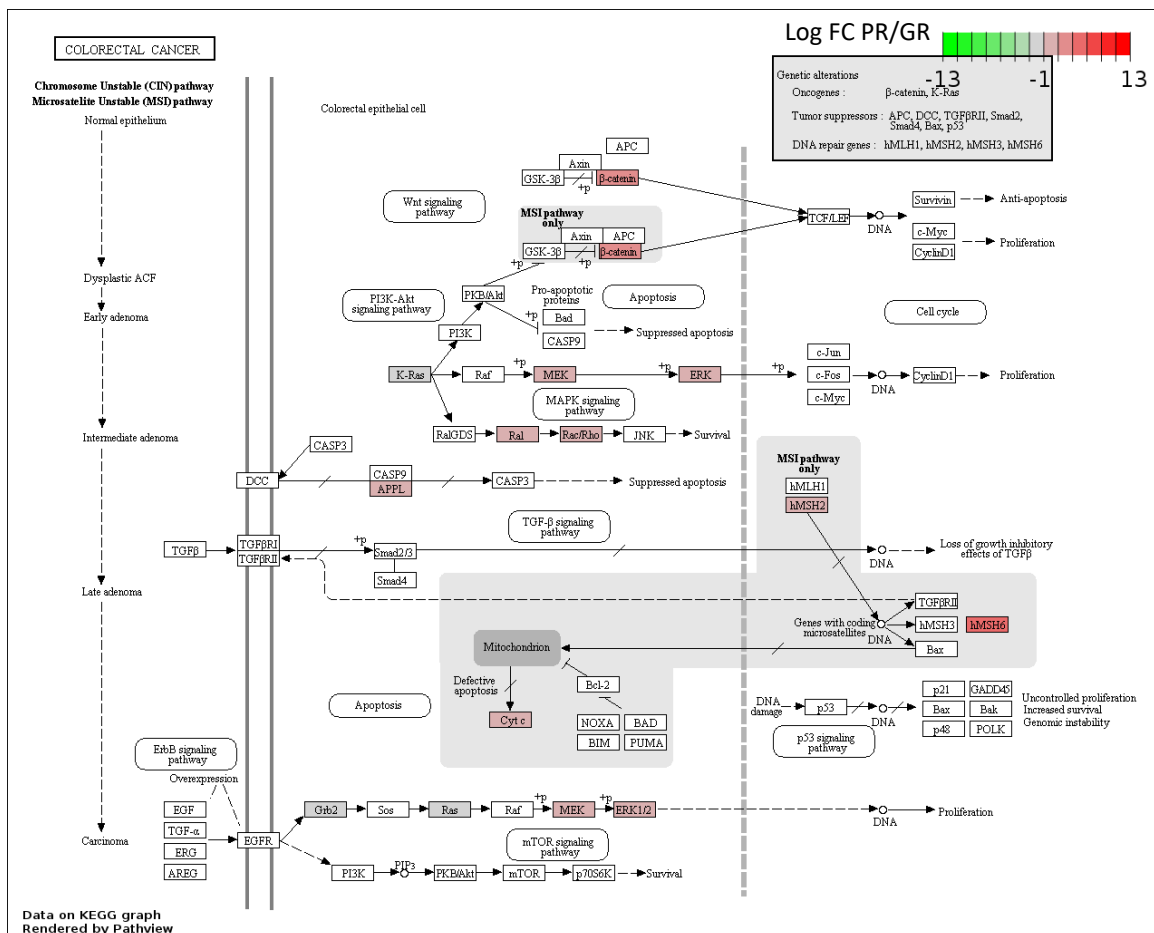

H)

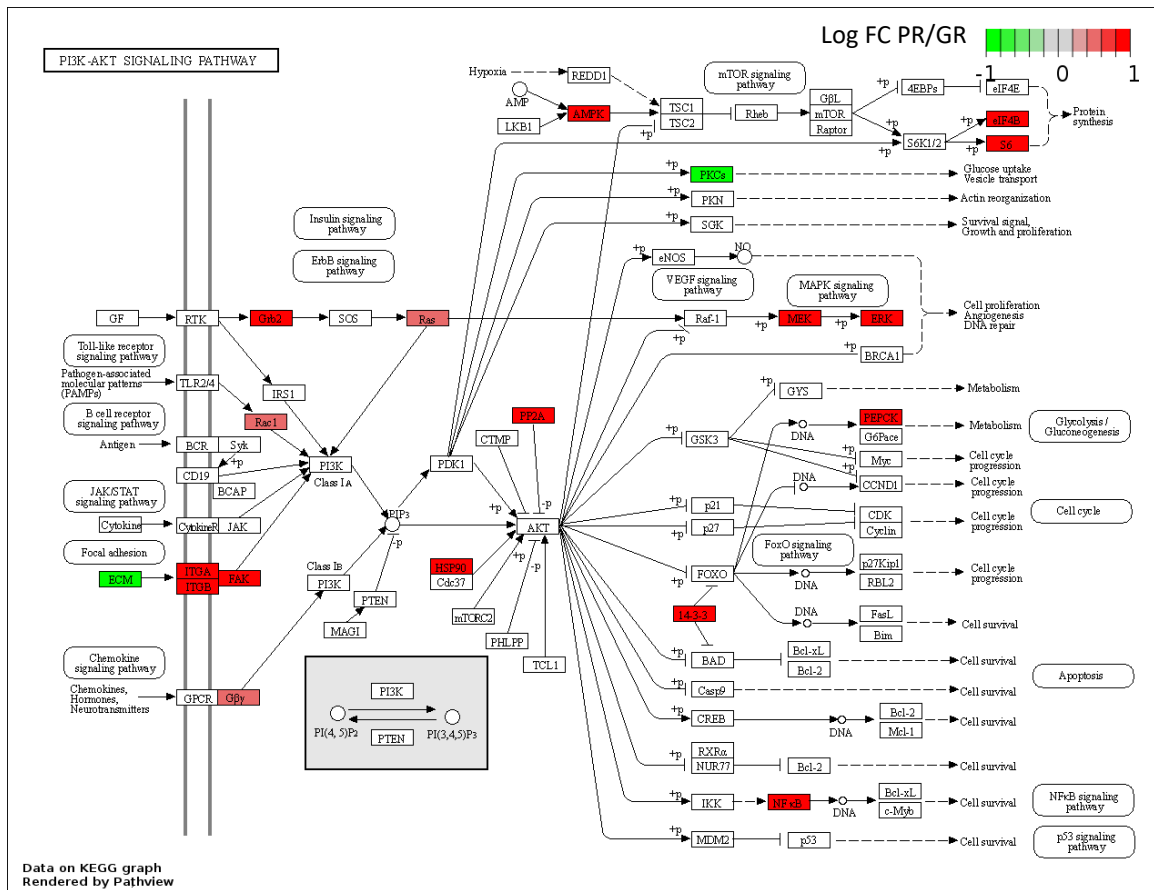

I)

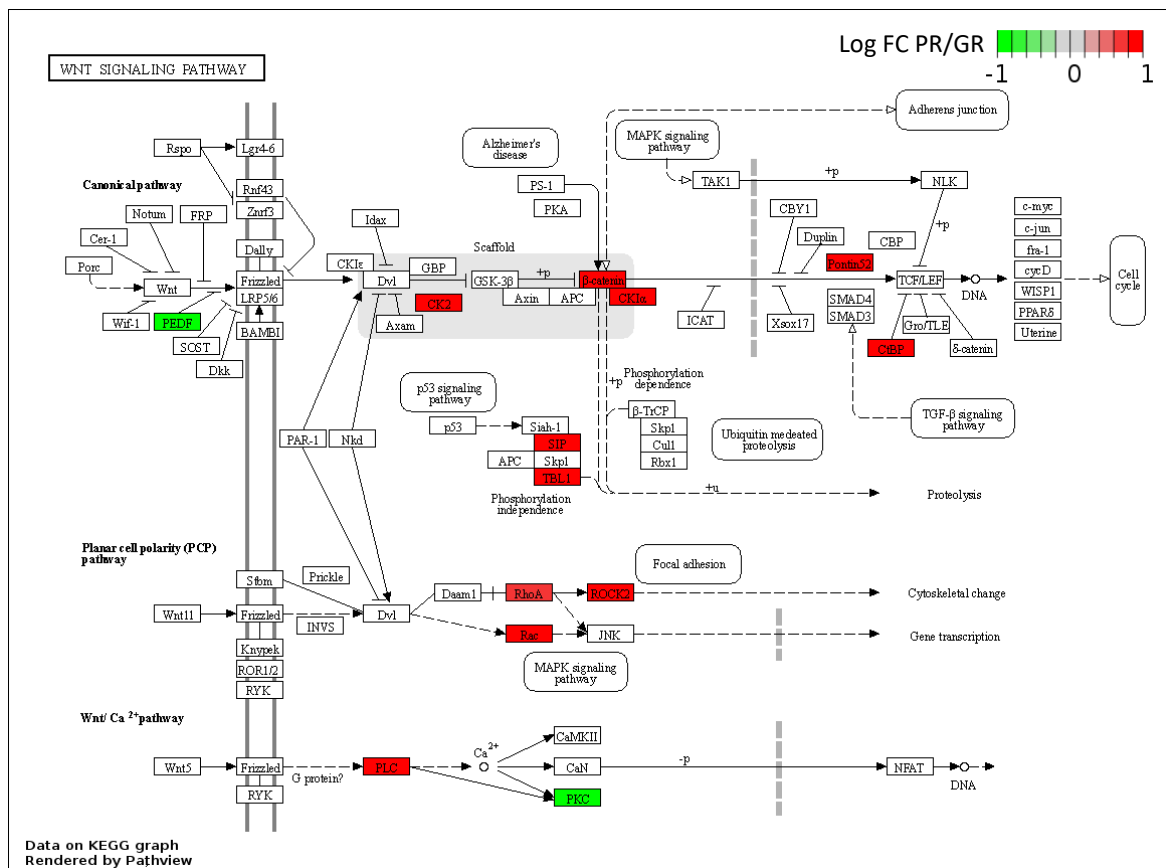

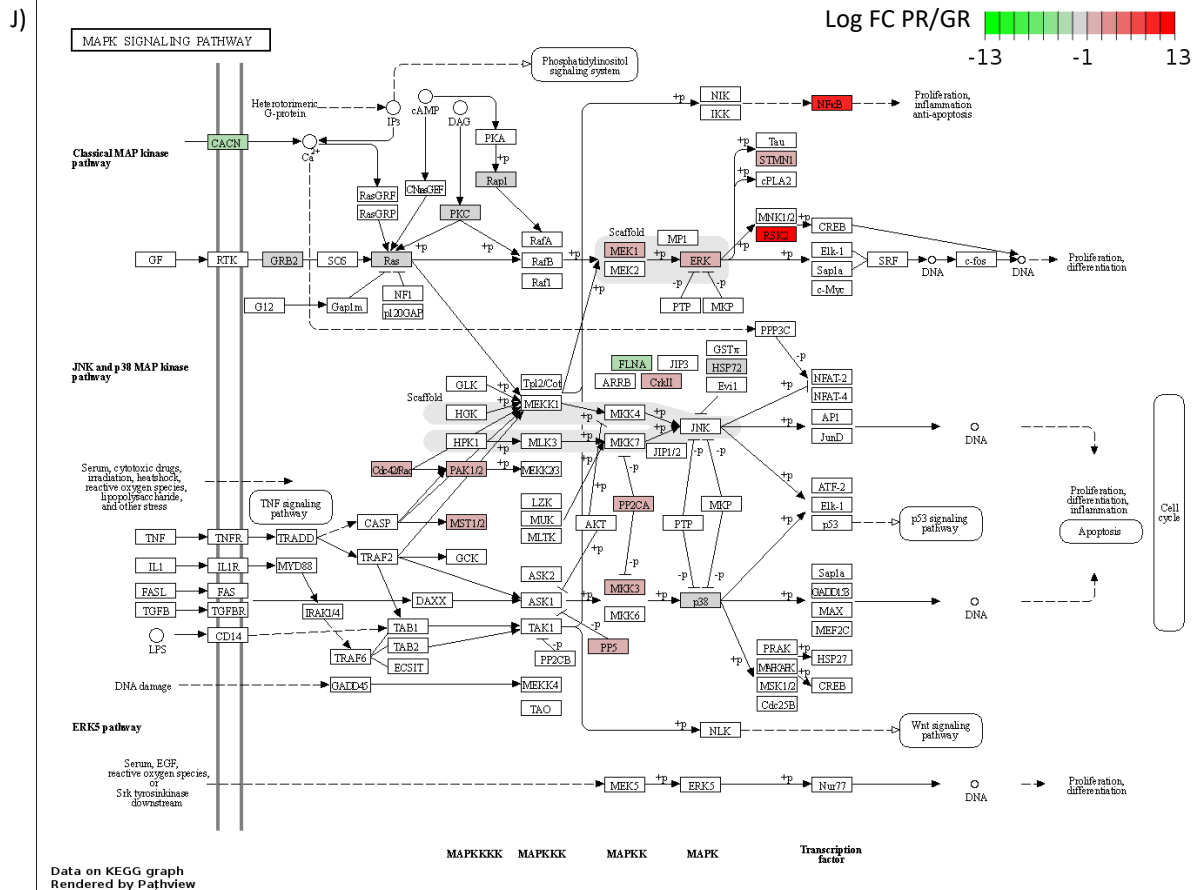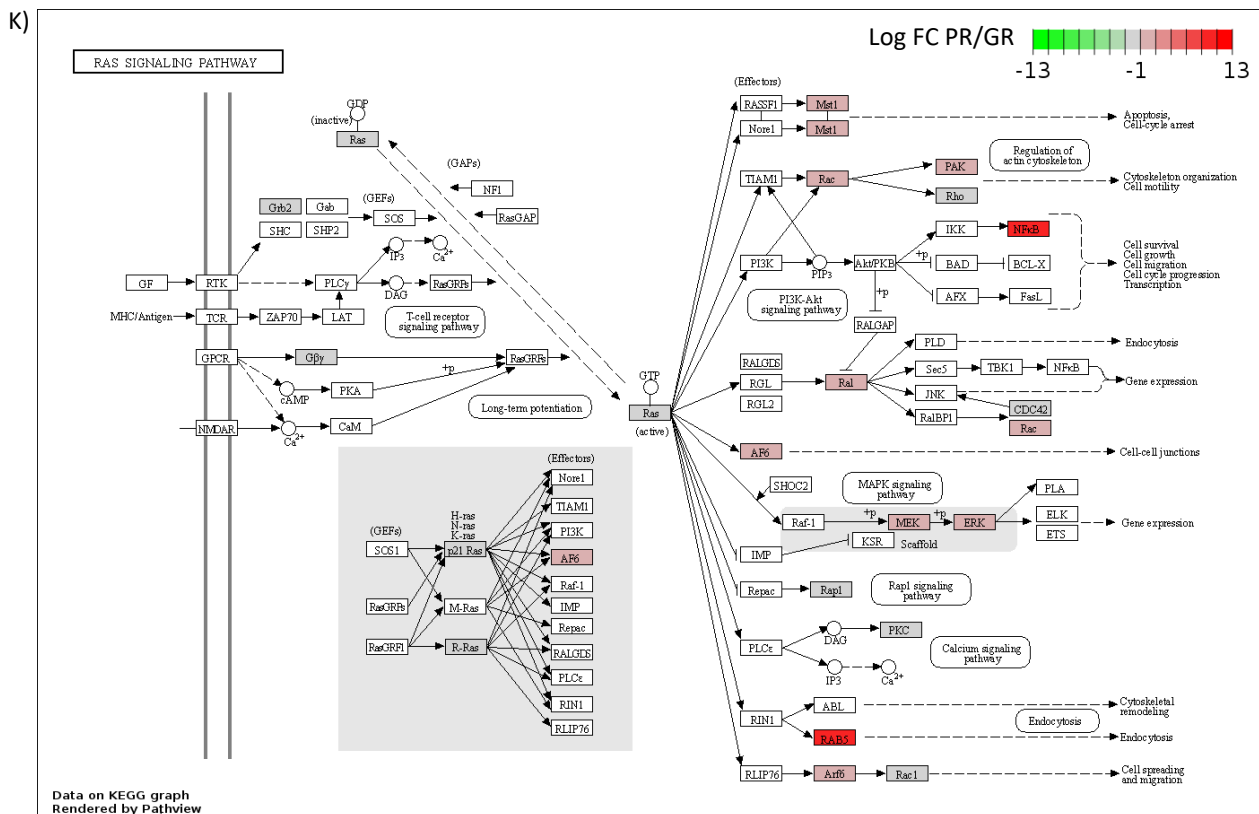

L)

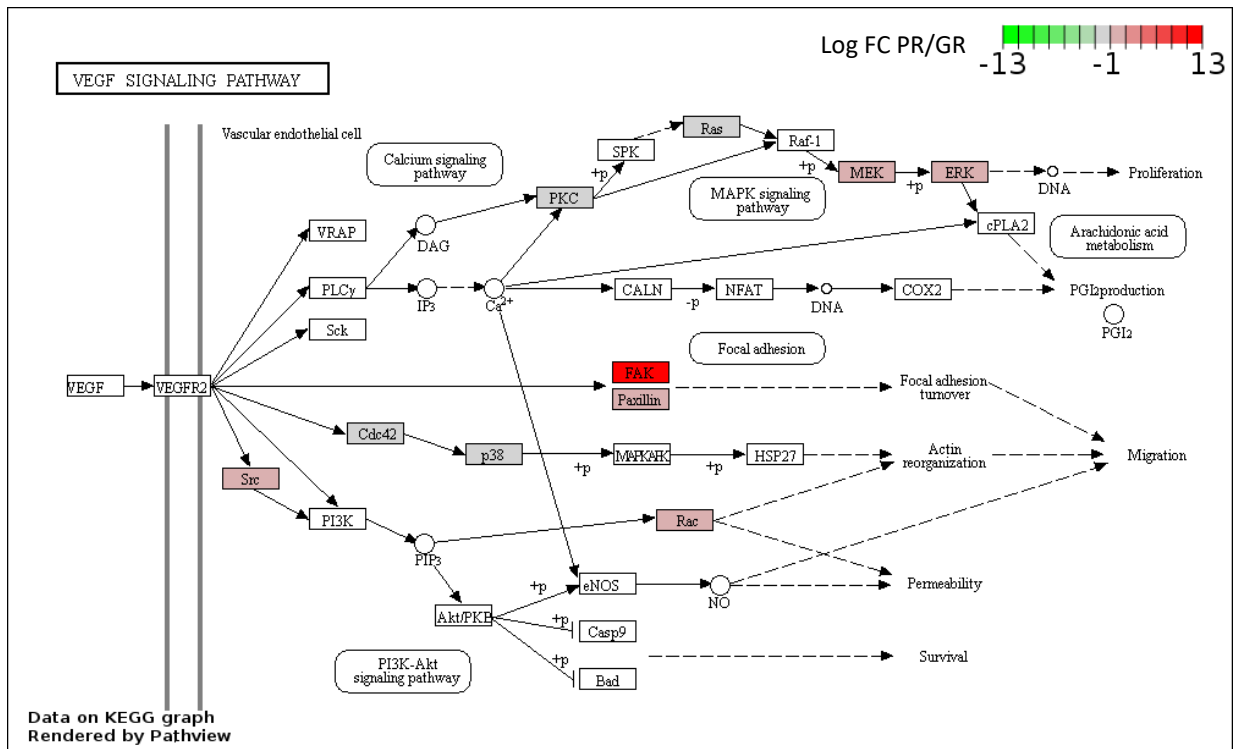

M)

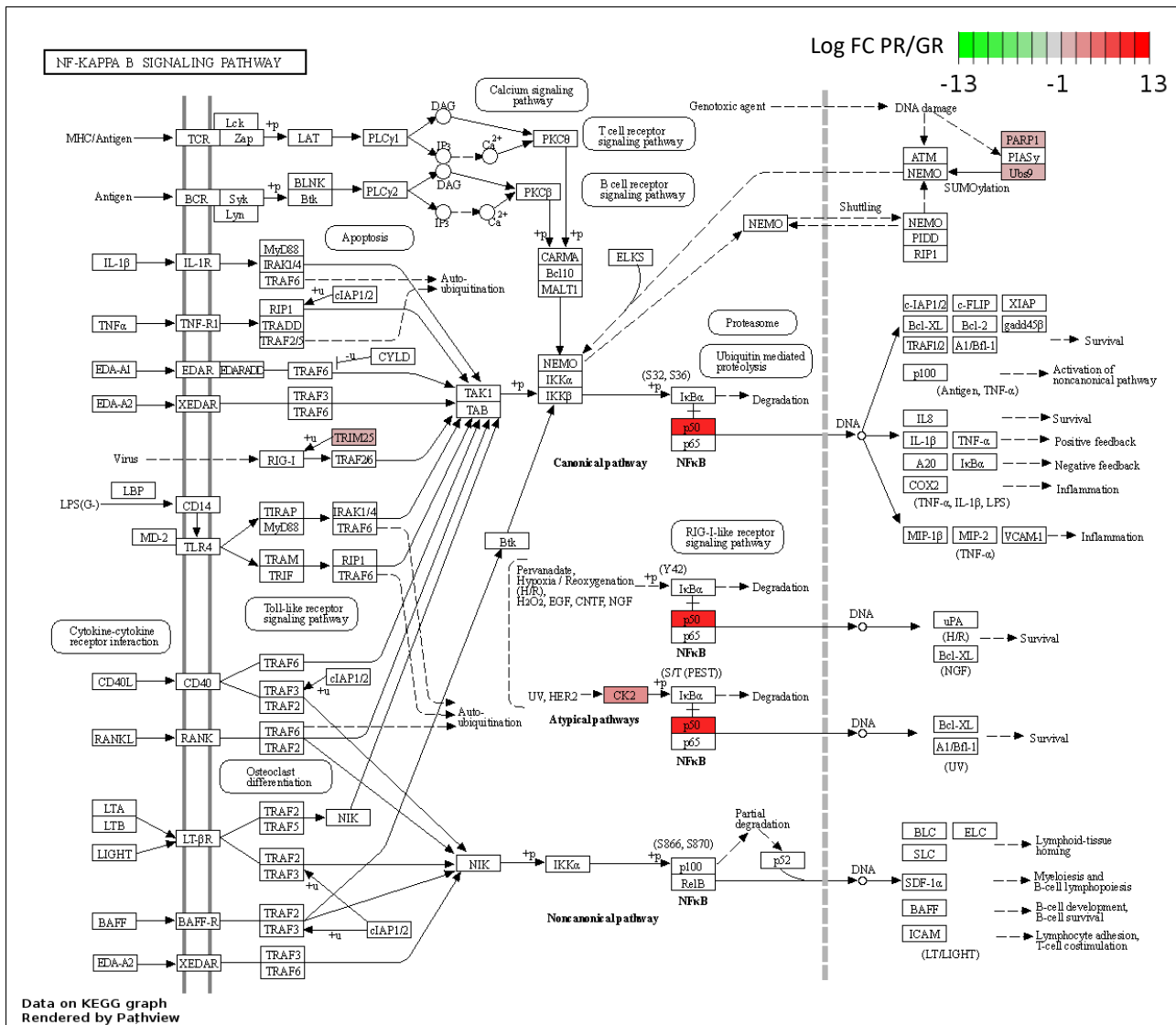

Supplement: Supplementary Figure 5 — Chosen the most enriched KEGG pathways connected with DAPs. (A) - Ribosome; (B) - Proteasome; (C) - ECM-receptor interaction; (D) - Proteoglycans in cancer; (E) - Focal adhesion; (F) - Complement and coagulation cascade; (G) - Colorectal cancer; (H) - PI3K-AKT signaling pathway; (I) - WNT signaling pathway; (J) - MAPK signaling pathway; (K) - RAS signaling pathway; (L) - VEGF signaling pathway; (M) - NF-KAPPA B signaling pathway. [file Image_5.pdf]

A)

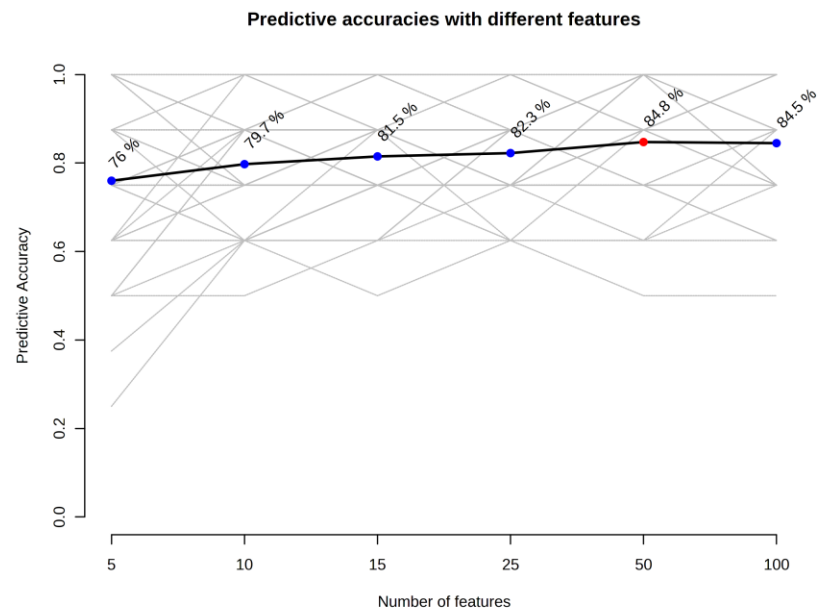

D)

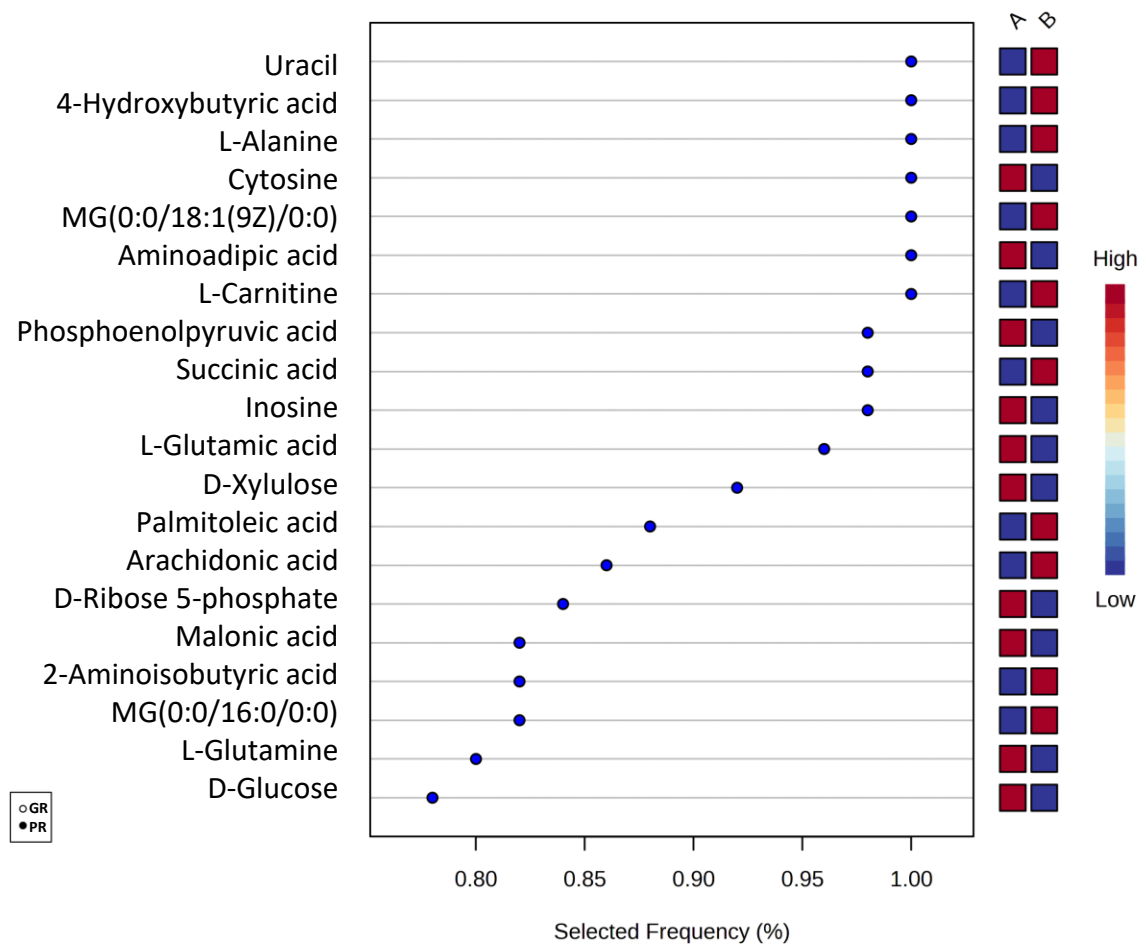

B)

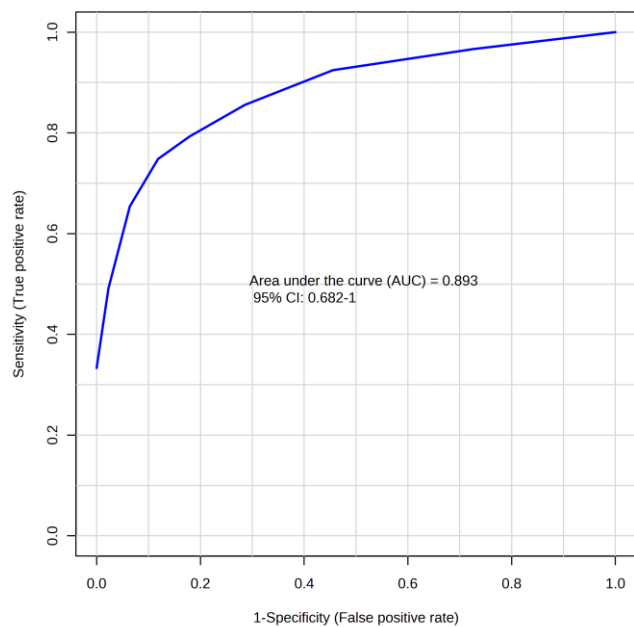

C)

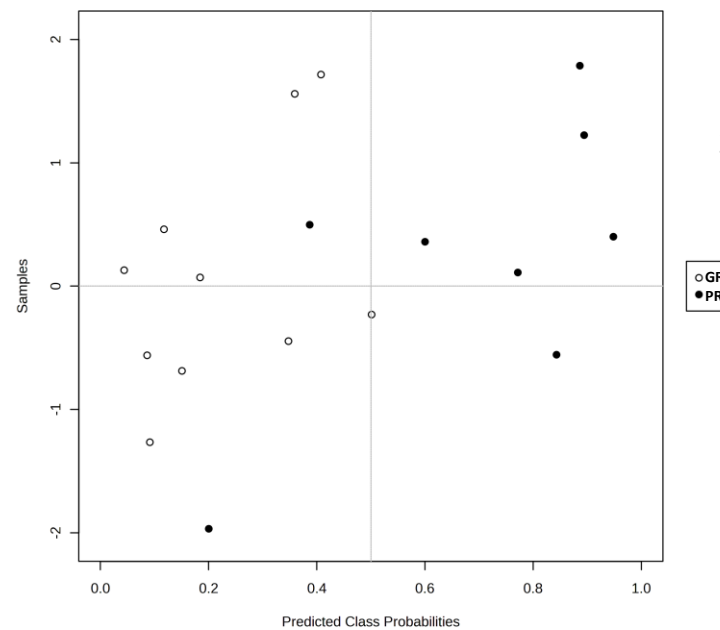

Supplement: Supplementary Figure 6 — Biomarker prediction by multivariate ROC curve analysis based on metabolomic features. (A) - The predictive accuracies of 6 different biomarker models; For the 50-feature panel, the red dot indicates the highest accuracy.; (B) - ROC curve for a chosen biomarker model with the highest accuracy; (C) - The predicted class probabilities for each sample (GR vs. PR); (D) - The top 20 potential proteomic biomarkers predicted based on how frequently they were chosen for cross-validation. [file Image_6.pdf]

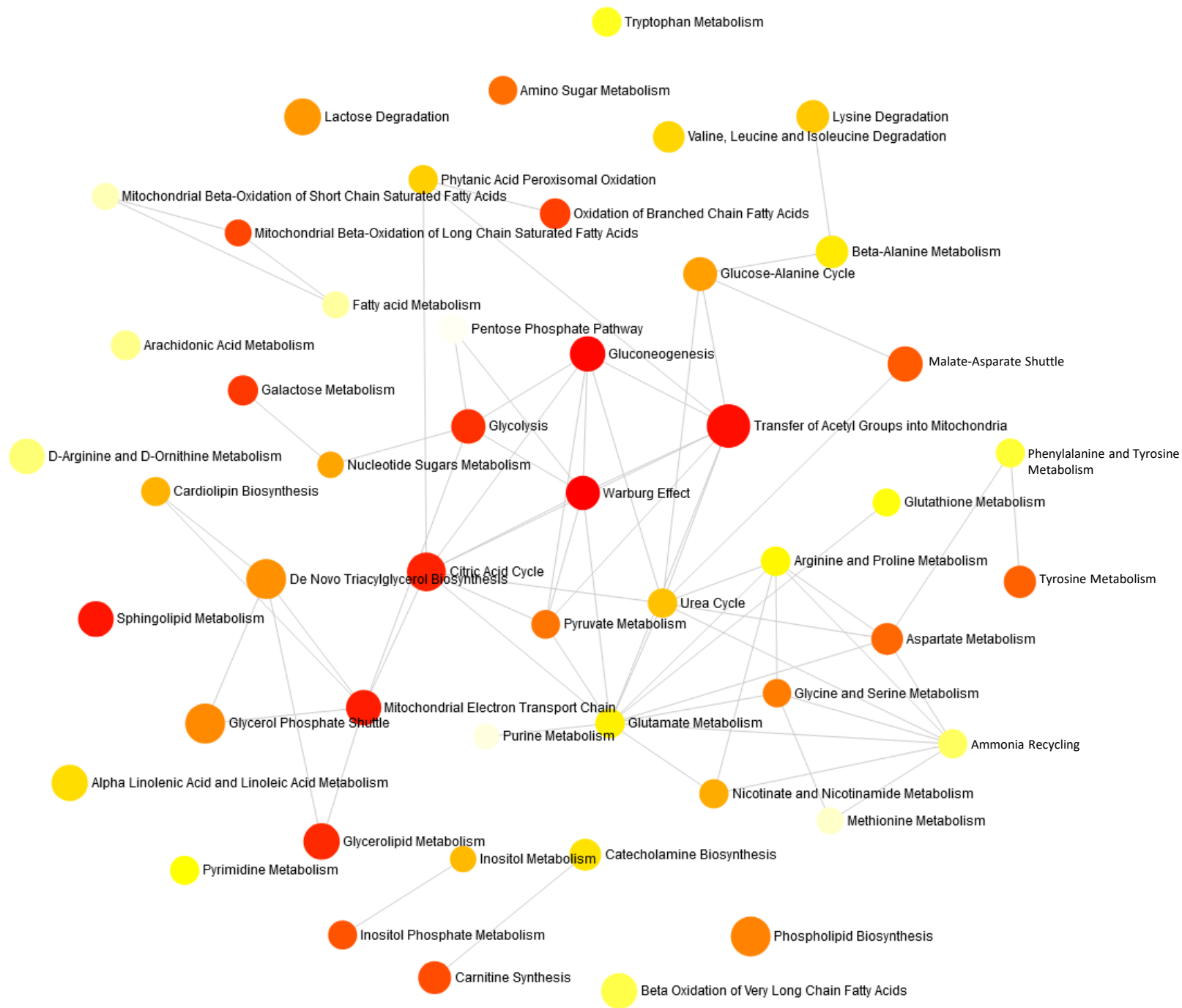

Supplement: Supplementary Figure 7 — Network view of significantly enriched metabolic pathways (FDR < 0.05) associated with DAMs based on quantitative enrichment analysis using KEGG database. [file Image_7.pdf]

[illegible]



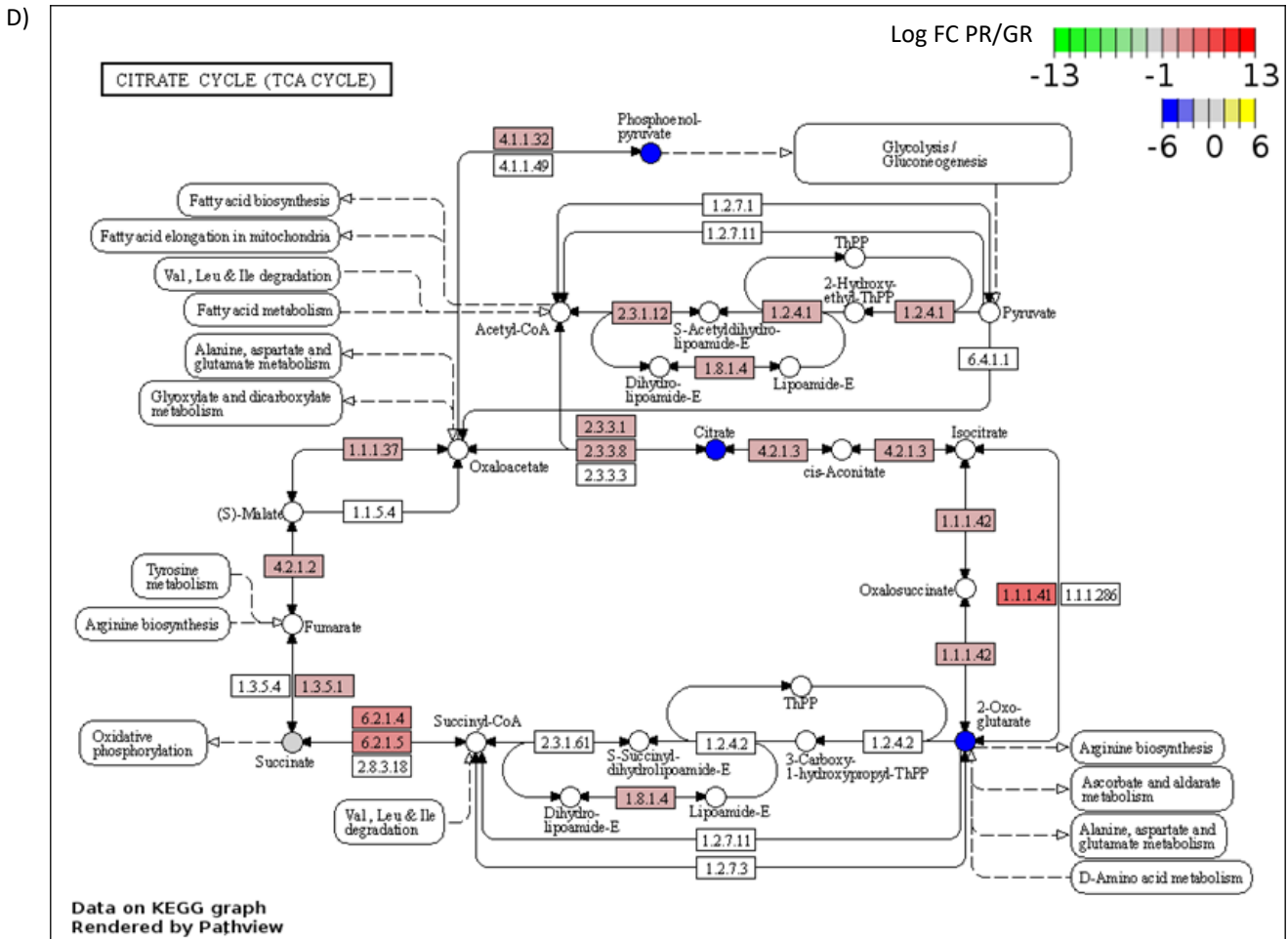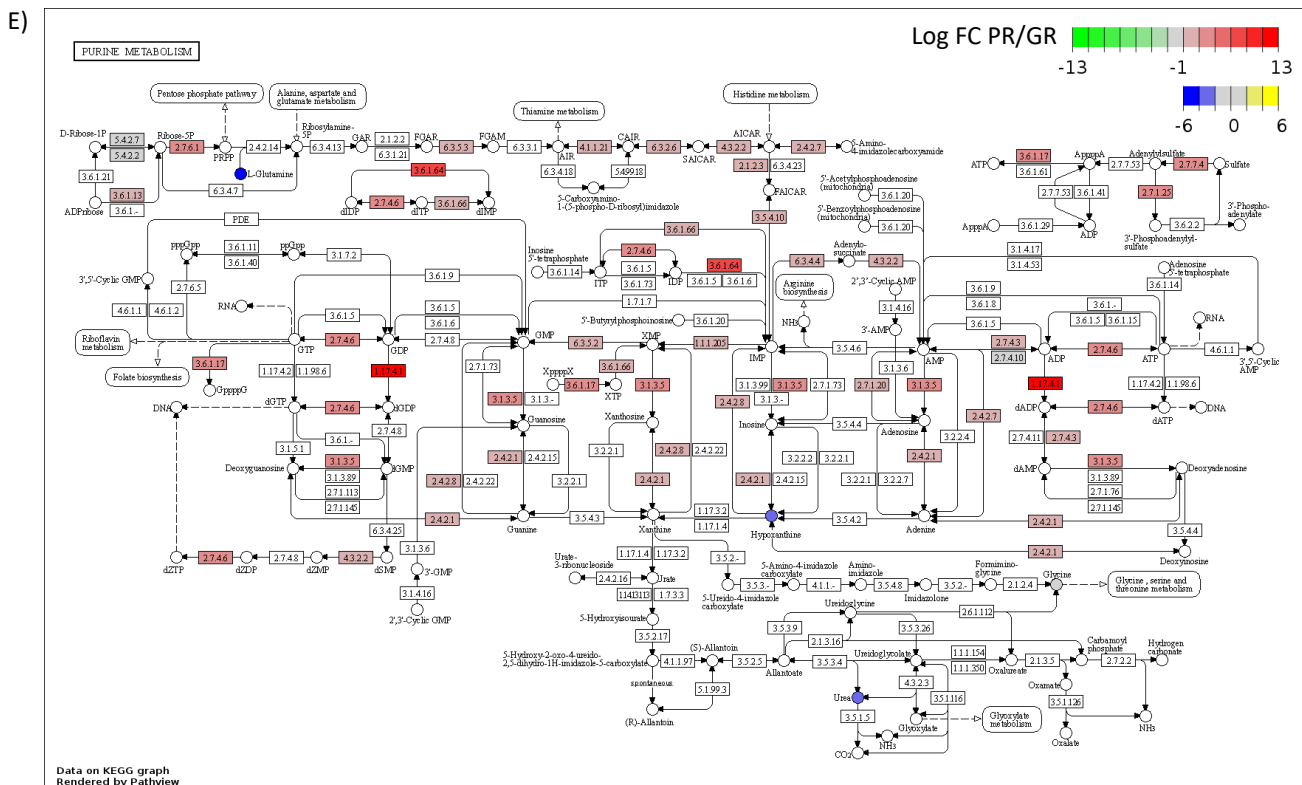

F)

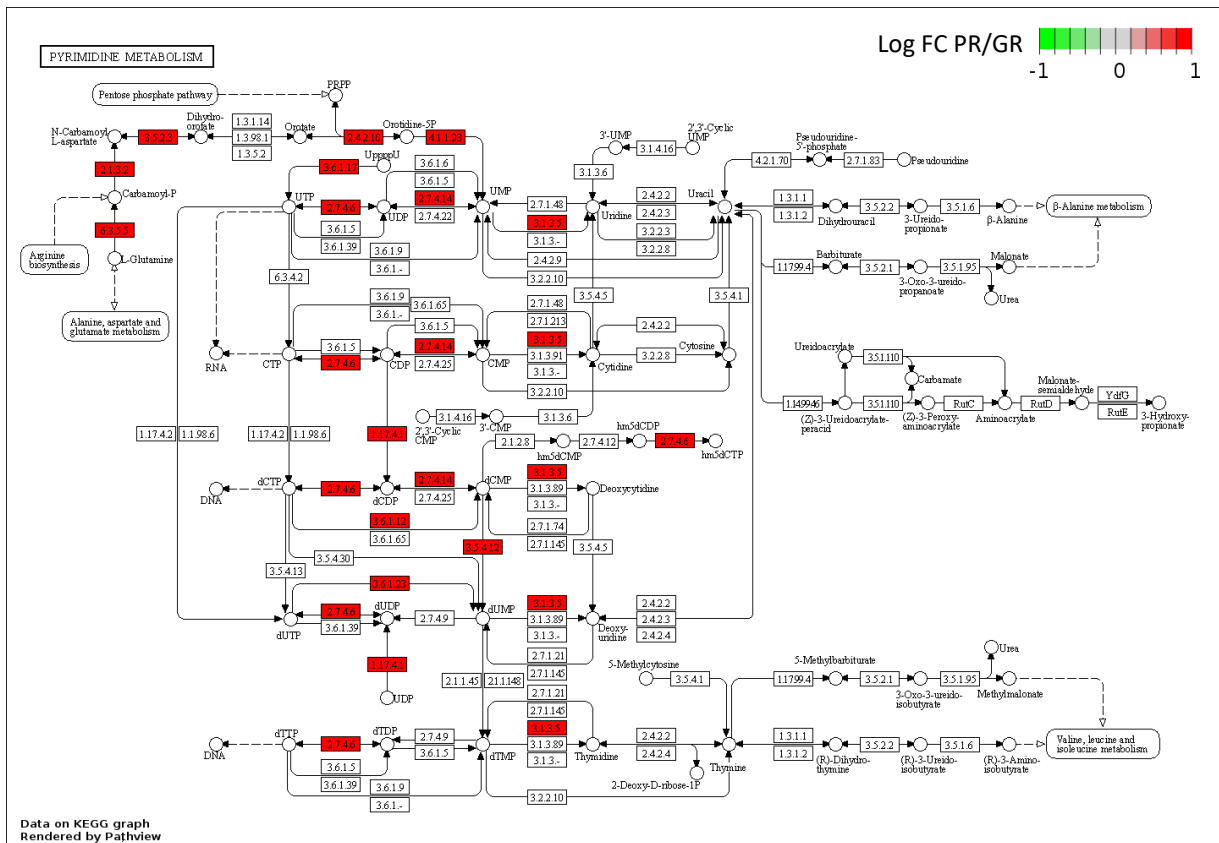

G)

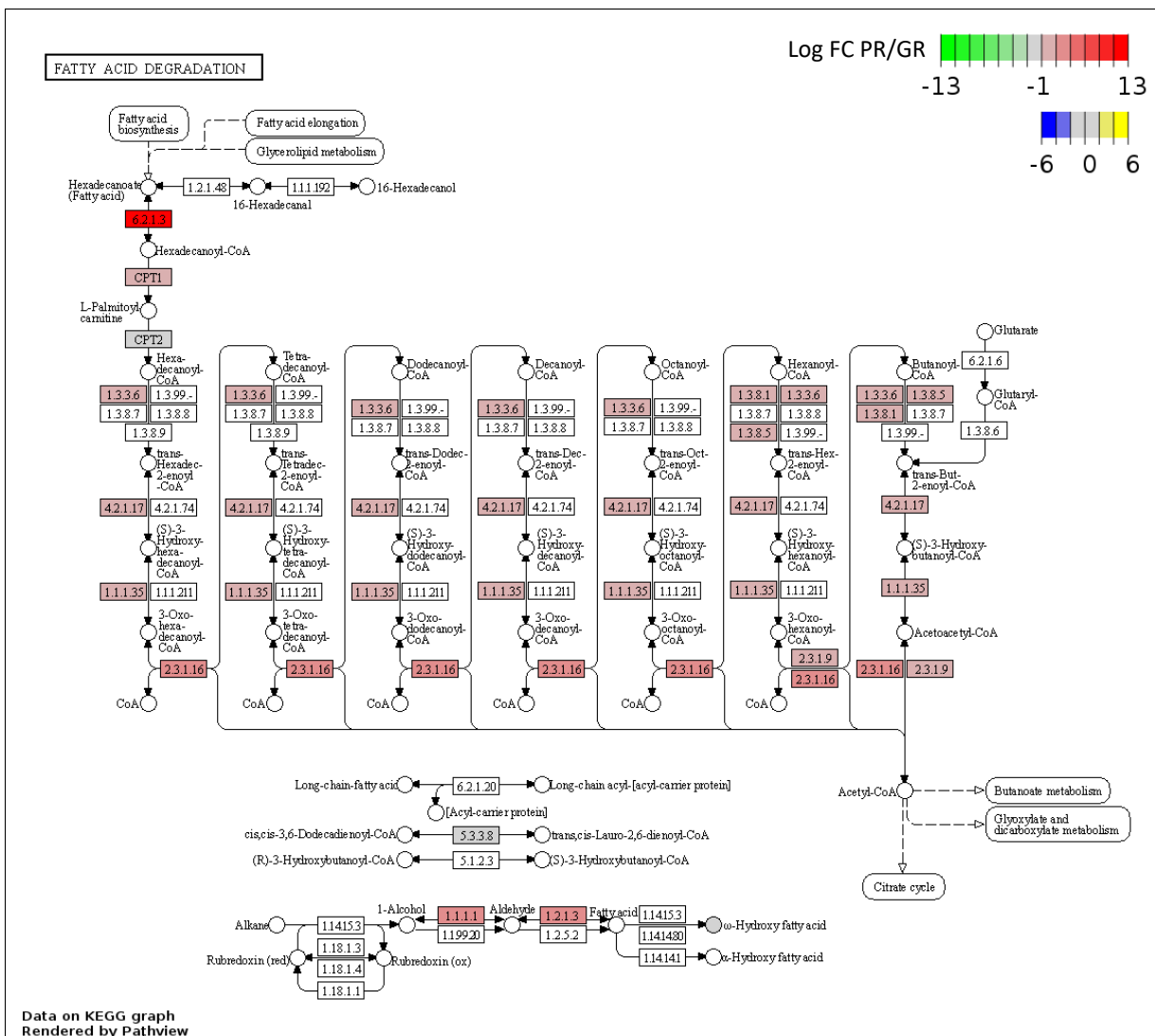

Supplement: Supplementary Figure 8 — Chosen the most enriched KEGG pathways connected with DAPs and DAMs. (A) - Glycolysis/gluconeogenesis; (B) - Pyruvate metabolism; (C) - Pentose phosphate pathway; (D) - Citrate cycle; (E) - Purine metabolism; (F) - Pyrimidine metabolism; (G) - Fatty acid degradation. [file Image_8.pdf]

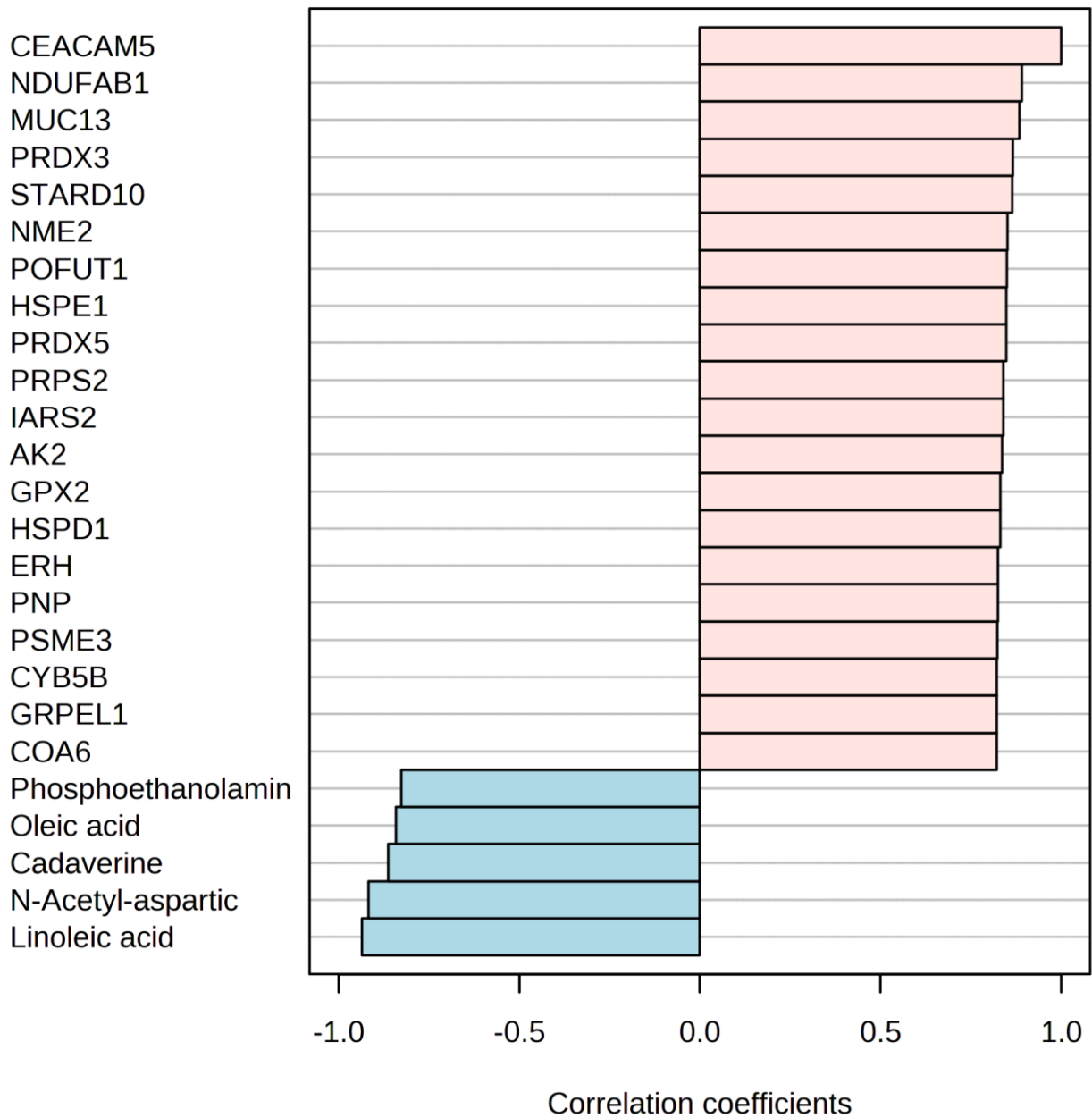

Supplement: Supplementary Figure 9 — The TOP 25 compounds correlated with CEACAM5 based on Pearson’s correlation analysis. [file Image_9.pdf]

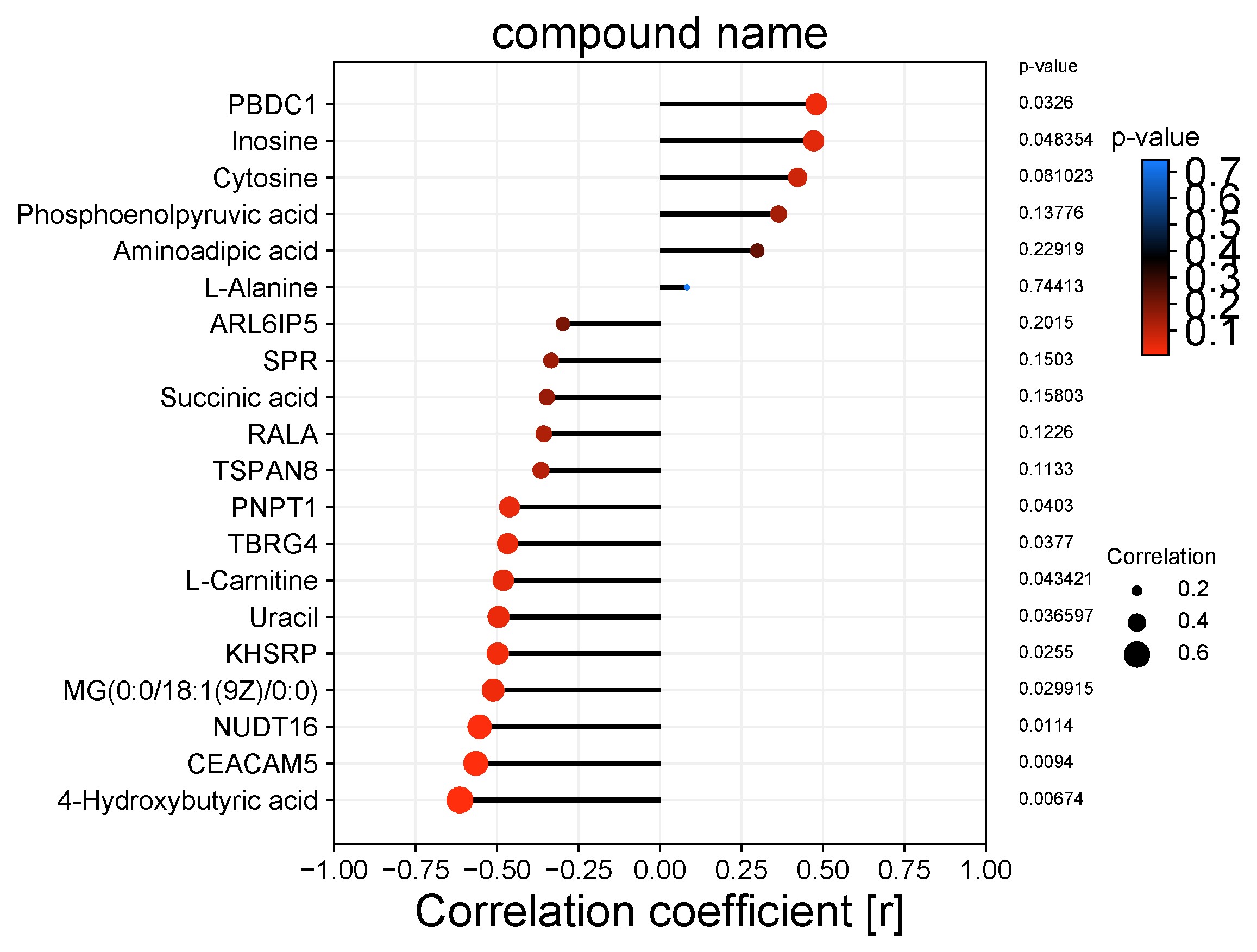

Supplement: Supplementary Figure 10 — The results of Pearson’s correlation analysis of the Top-10 DAPs and DAMs correlated with radiation dose in all patient groups. [file Image_10.jpeg]
