# Supplementary material for: Proteomic and metabolomic signatures of rectal tumor discriminate patients with different responses to preoperative radiotherapy
Source: Front Oncol. 2024 Feb 12;14:1323961. doi: 10.3389/fonc.2024.1323961 (PMC10896604; doi:10.3389/fonc.2024.1323961)
Supplement: Supplementary Figure 3 — Biomarker prediction by multivariate ROC curve analysis based on proteomic features. (A) - The predictive accuracies of 6 different biomarker models; For the 50-feature panel, the red dot indicates the highest accuracy.; (B) - ROC curve for a chosen biomarker model with the highest accuracy; (C) -The predicted class probabilities for each sample (GR vs. PR); (D) - The top 20 potential proteomic biomarkers predicted based on how frequently they were chosen for cross-validation. [file Image_3.pdf]

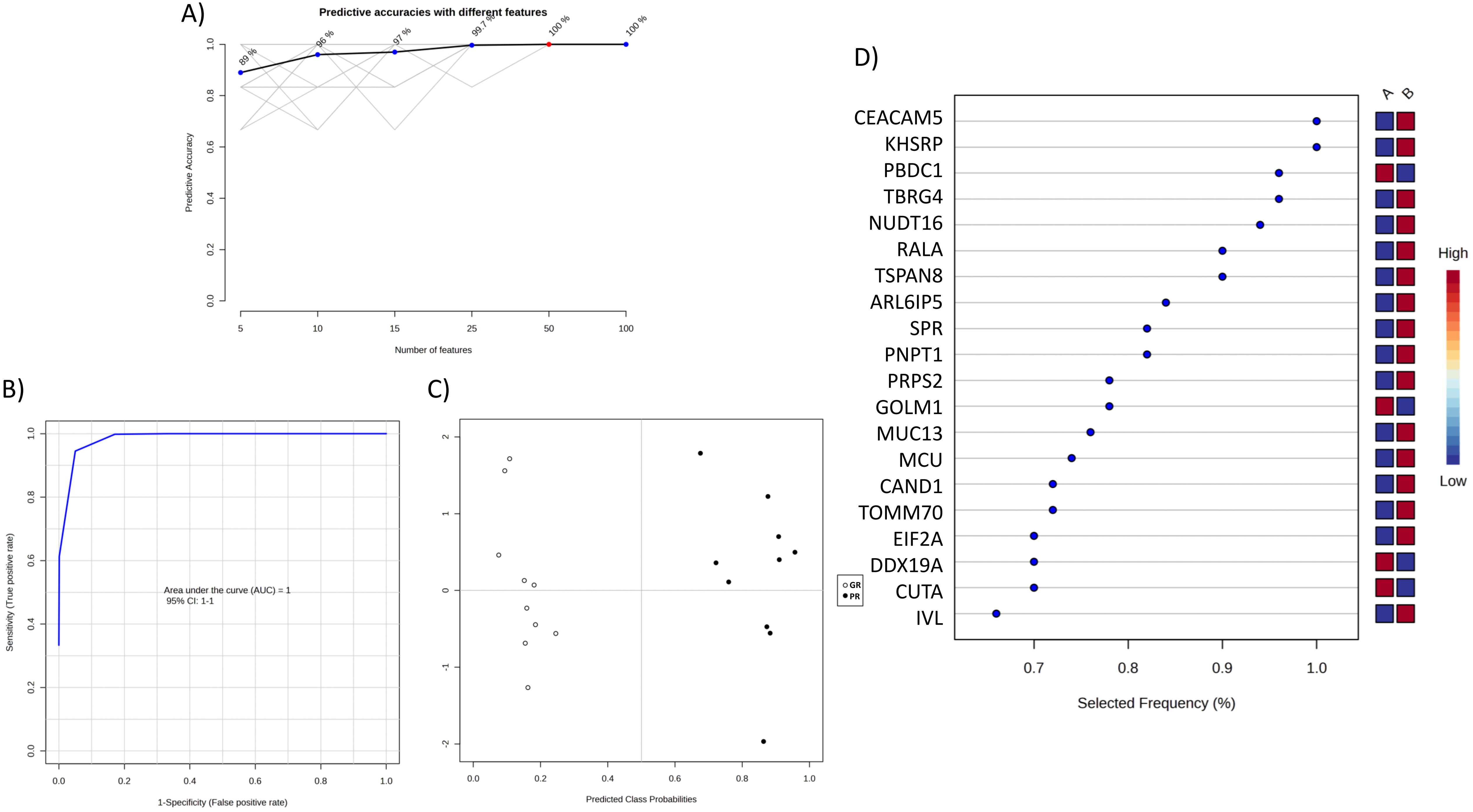
A)
Predictive accuracies with different features
89 %
96 %
97 %
99.7 %
100 %
100 %
Predictive Accuracy
0.0
0.2
0.4
0.6
0.8
1.0
5
10
15
25
50
100
Number of features
B)
Sensitivity (True positive rate)
Area under the curve (AUC) = 1
95% CI: 1-1
0.0
0.2
0.4
0.6
0.8
1.0
1-Specificity (False positive rate)
C)
2
1
0
-1
-2
GR
PR
Predicted Class Probabilities
D)
A
B
CEACAM5
KHSRP
PBDC1
TBRG4
NUDT16
RALA
TSPAN8
ARL6IP5
SPR
PNPT1
PRPS2
GOLM1
MUC13
MCU
CAND1
TOMM70
EIF2A
DDX19A
CUTA
IVL
0.7
0.8
0.9
1.0
Selected Frequency (%)
High
Low
