# Supplementary material for: Proteomic and metabolomic signatures of rectal tumor discriminate patients with different responses to preoperative radiotherapy
Source: Front Oncol. 2024 Feb 12;14:1323961. doi: 10.3389/fonc.2024.1323961 (PMC10896604; doi:10.3389/fonc.2024.1323961)
Supplement: Supplementary Figure 4 — Overrepresented pathways involved in the immune response connected with DAPs upregulated in PR. (A) - bubble plots of the TOP12 enriched immunological processes and functions revealed using the GO terms; (B) - chosen significantly overrepresented pathways involved in the immune response. [file Image_4.pdf]

A)

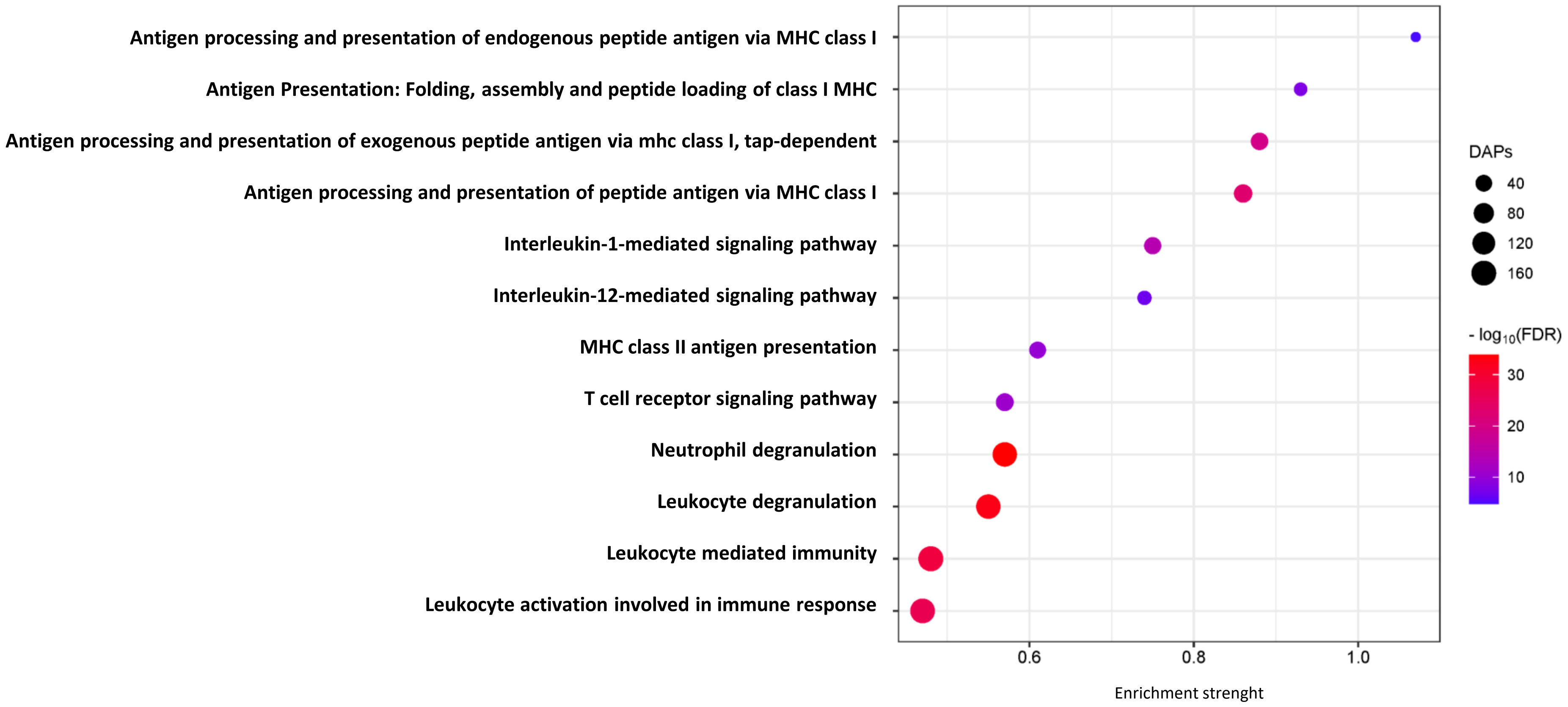
Antigen processing and presentation of endogenous peptide antigen via MHC class I
Antigen Presentation: Folding, assembly and peptide loading of class I MHC
Antigen processing and presentation of exogenous peptide antigen via mhc class I, tap-dependent
Antigen processing and presentation of peptide antigen via MHC class I
Interleukin-1-mediated signaling pathway
Interleukin-12-mediated signaling pathway
MHC class II antigen presentation
T cell receptor signaling pathway
Neutrophil degranulation
Leukocyte degranulation
Leukocyte mediated immunity
Leukocyte activation involved in immune response
DAPs
40
80
120
160
- log10(FDR)
30
20
10
0.6
0.8
1.0
Enrichment strenght

B)

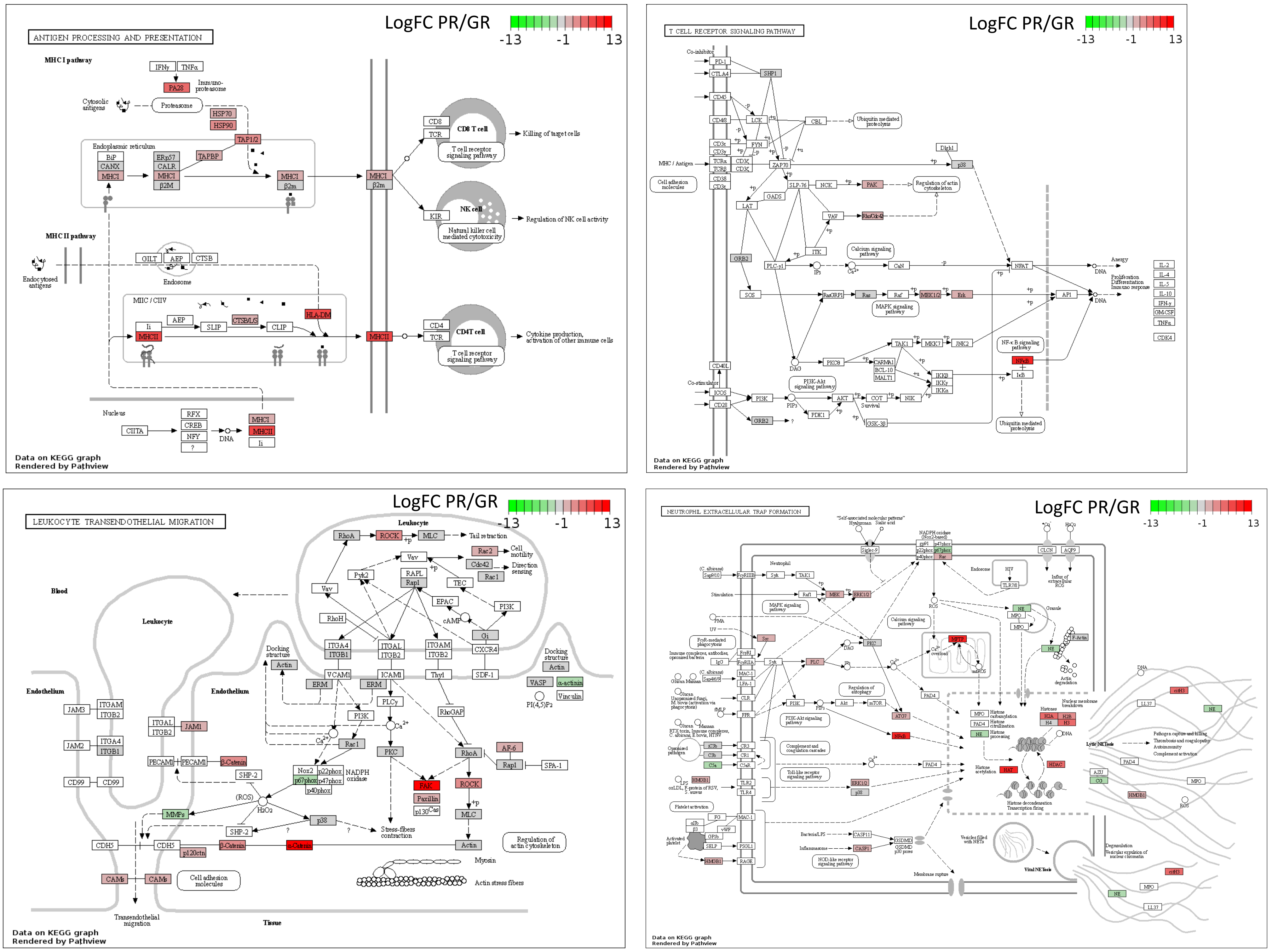
ANTIGEN PROCESSING AND PRESENTATION
LogFC PR/GR
-13
-1
13
MHC I pathway
MHC II pathway
CD8 T cell
NK cell
CD4T cell
Killing of target cells
Regulation of NK cell activity
Cytokine production, activation of other immune cells
Data on KEGG graph
Rendered by Pathview
T CELL RECEPTOR SIGNALING PATHWAY
LogFC PR/GR
-13
-1
13
Data on KEGG graph
Rendered by Pathview
LEUKOCYTE TRANSENDOTHELIAL MIGRATION
LogFC PR/GR
-13
-1
13
Blood
Leukocyte
Endothelium
Tissue
Transendothelial migration
Data on KEGG graph
Rendered by Pathview
NEUTROPHIL EXTRACELLULAR TRAP FORMATION
LogFC PR/GR
-13
-1
13
Data on KEGG graph
Rendered by Pathview
